# Shallow conductance decay along the *heme* array of a single tetraheme protein wire†

**DOI:** 10.1039/d4sc01366b

**Published:** 2024-07-03

**Authors:** Kavita Garg, Zdenek Futera, Xiaojing Wu, Yongchan Jeong, Rachel Chiu, Varun Chittari Pisharam, Tracy Q. Ha, Albert C. Aragonès, Jessica H. van Wonderen, Julea N. Butt, Jochen Blumberger, Ismael Díez-Pérez

**Affiliations:** a Department of Chemistry, Faculty of Natural, Mathematical & Engineering Sciences, King's College London, Britannia House 7 Trinity Street London SE1 1DB UK ismael.diez_perez@kcl.ac.uk; b Department of Physics and Astronomy, Thomas Young Centre, University College London Gower Street London WC1E 6BT UK j.blumberger@ucl.ac.uk; c School of Chemistry, School of Biological Sciences, University of East Anglia Norwich Research Park Norwich NR4 7TJ UK J.Butt@uea.ac.uk; d Faculty of Science, University of South Bohemia Branisovska 1760 370 05 Ceske Budejovice Czech Republic; e Departament de Ciència de Materials i Química Física, Universitat de Barcelona, Institut de Química Teòrica i Computacional (IQTC) Marti i Franquès 1 08028 Barcelona Spain

## Abstract

Multiheme cytochromes (MHCs) are the building blocks of highly conductive micrometre-long supramolecular wires found in so-called electrical bacteria. Recent studies have revealed that these proteins possess a long supramolecular array of closely packed *heme* cofactors along the main molecular axis alternating between perpendicular and stacking configurations (TST = T-shaped, stacked, T-shaped). While TST arrays have been identified as the likely electron conduit, the mechanisms of outstanding long-range charge transport observed in these structures remain unknown. Here we study charge transport on individual small tetraheme cytochromes (STCs) containing a single TST *heme* array. Individual STCs are trapped in a controllable nanoscale tunnelling gap. By modulating the tunnelling gap separation, we are able to selectively probe four different electron pathways involving 1, 2, 3 and 4 *heme* cofactors, respectively, leading to the determination of the electron tunnelling decay constant along the TST *heme* motif. Conductance calculations of selected single-STC junctions are in excellent agreement with experiments and suggest a mechanism of electron tunnelling with shallow length decay constant through an individual STC. These results demonstrate that an individual TST motif supporting electron tunnelling might contribute to a tunnelling-assisted charge transport diffusion mechanism in larger TST associations.

## Abstract 2

Small tetraheme cytochromes (STCs) support efficient electron tunnelling suggesting tunnelling-assisted long-range charge transport in large multiheme structures.

## Graphical abstract

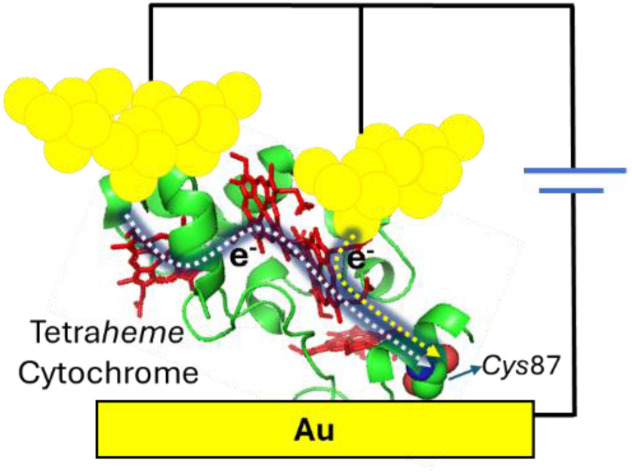

## Introduction

Nature has evolved numerous supramolecular conduits for efficient charge and energy transport. Prominent examples are bacterial nanowires, BN, from *Geobacter* or *Shewanella*, which stand out because of their remarkable charge transport capabilities. They are made of multiheme cytochrome (MHC) complexes that transport electrons over record distances from several tens of nanometres to a hundred microns.^1–4^ Protein nanowires produced by microbes or fabricated *in vitro* with microbe-inspired designs, are “green” electronic materials with potential for functionalization that can translate the outstanding electrical performance in nature to potential applications beyond those feasible with synthetic polymers. In particular, the natural ability of BN to interface with inorganic materials opens new prospects in the incorporation of BN onto electrodes, leading to a new technological panorama using bioelectronic interfaces as the active electrical components.^5–7^

**Fig. 1:**
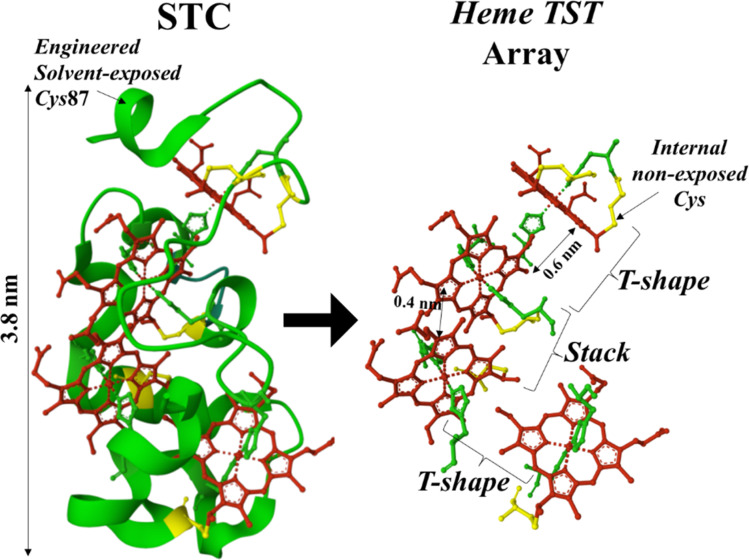
STC crystal structure (left panel) from PDB ID 1M1P indicating the position of the solvent-exposed engineered cysteine (*Cys*) residue at position 87. Right panel shows the TST-pairing array of the four *heme* cofactors in the STC structure. Each *heme* cofactor is axially coordinated by two histidine residues (green) and covalently attached to the peptide backbone (green ribbon) *via* internal (non-solvent exposed) *Cys* residues (yellow).

Among the family members of MHCs, STC (small tetraheme cytochrome, ∼12 kDa) from *Shewanella oneidensis* shuttles electrons from the cell metabolism delivered by the inner-membrane CymA cytochrome to the porin–cytochrome complex MtrCAB in the outer-membrane. STC constitutes one of the most elementary multiheme structures with four *heme* cofactors spanning the full length (∼3.8 nm) of the STC backbone (Fig. 1, left panel). The array of the 4 *heme* cofactors follows a structure with alternating perpendicular and stacking *heme* configurations (Fig. 1, right panel) that we refer to as TST (T-shaped pair, stacked pair, T-shaped pair). STC brings the opportunity to study the charge transport characteristics in a single TST motif, which is found in larger MHC complexes, *e.g.*, MtrAB and OmcS,^8,9^ as extended (TST)_*n*_ arrays in different species of bacteria. Building an atomic scale model of charge transport in a TST motif would revolutionise our understanding of biological electron transport as well as open to the design of a new class of conducting polymers with enormous technological impact.

In the last three decades, the field of single-molecule electronics^10^ has established robust methodologies to characterize the conductance of an individual molecule trapped in a nanoscale gap.^11–14^ The latter approaches have now extended to individual proteins and made extensive contributions to advancing the field of protein electronics where research has shown; (1) outstanding conductivity displayed by redox as well as non-redox proteins,^15–20^ and (2), the relevance of engineering the electrode/protein interface to achieve both adequate protein orientation in a junction and optimal electrode/protein electrical communication (coupling).^7,21–25^ Pioneering research on charge transport in MHCs integrated in a vacuum solid-state device demonstrated that MHC-based monolayers sandwiched between two gold electrodes provide high, long-range electron conduction equivalent to that of conjugated molecular backbones, and poor temperature-dependence of conductance, suggesting coherent charge transport.^25^ The latter has prompted intense discussions around the mechanisms of charge transport that could explain the main electrical signatures observed in multiheme-based BN.

Here we investigate the charge transport behaviour of an STC in an aqueous environment under ambient conditions using a single-protein junction approach. STC is a highly conserved soluble protein among *Shewanella* species bearing a single TST motif (Fig. 1). To measure single-protein conductance in a STC protein, individual STCs are trapped in a tunnelling junction of a scanning tunnelling microscope (STM) immersed in near physiological aqueous electrolyte under ambient conditions. Such an approach has been widely used for the conductance characterization of metalloporphyrins.^26–29^ In the past decade, we and others have also exploited these approaches to characterize single-protein charge transport in a plethora of redox as well as non-redox proteins.^15,19,24,30–33^ Our single-STC junction experiments characterize for the first time the conductance of an individual MHC in a wet (aqueous) medium. Specific interactions of the STC protein with the STM junction electrodes allows for the analysis of the distance dependence of conductance, revealing a rather low (∼2 nm^−1^) electron tunnelling decay constants along the main protein axis. Both STM-based single-protein charge transport experiments and computational calculations of modelled STC junctions under defined protein orientations, support the obtained experimental conductance values and decay constant. The results suggest an efficient low-barrier tunnelling mechanism through an individual TST *heme* motif, which could contribute to a tunnelling-assisted electron diffusion in long MHC-based structures.^34^

## Results and discussion

### Preparation of STC-modified electrodes

**Fig. 2:**
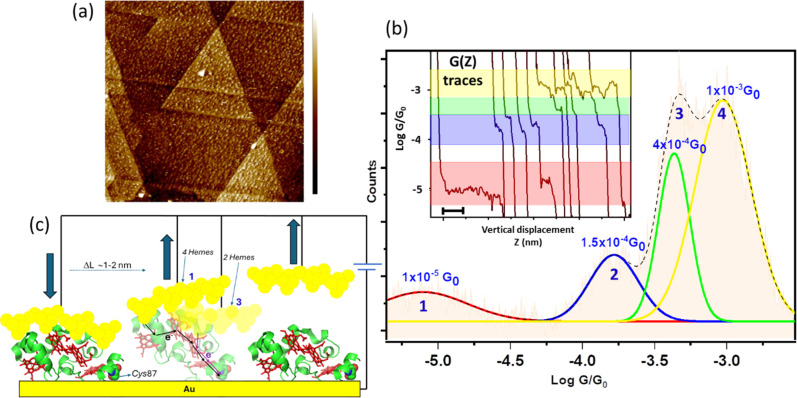
(a) 400 × 400 nm^2^ STM image of *Cys*87-modified STC proteins on Au surface. Each bright spot in the image corresponds to an individual STC. Characteristic triangulated atomically flat Au(111) terraces are >200 nm wide. Colour legend on the right spans from 0 to 1.2 nm. (b) Semi-log scale conductance (*G*) histogram accumulating 1350 selected *G*(*z*) traces (representative ones in the inset) at an applied 200 mV bias voltage. Individual Gaussian fits of the peaks (coloured solid lines) are used to extract the maxima values corresponding to the average conductance of each of the four features. The dotted line (----) is a cumulative plot including all the Gaussian peaks for visual guidance. Figure inset shows illustrative individual *G*(*z*) traces displaying plateau features used to build the 1D histogram. The *X*-axis scale bar at the left-bottom corner corresponds to 0.6 nm. (c) Pictorial representation of the STM break-junction process showing two possible scenarios leading to the low *G* (peak 1 in (b)) and high *G* (peak 3 in (b)) involving 4 and 2 *hemes* in the pathway, respectively, where STM tip asperities (represented as yellow balls) contact different points of the protein surface during different pulling cycles of the anchored (*via Cys*(thiol)) STC on the Au substrate.

To generate a stable electrode/protein interaction, we produced a genetically modified STC with a solvent exposed *Cys* residue at the highly solvent-exposed *Ser*87 native position (Fig. 1, left panel). The STC protein was purified from *Shewanella oneidensis* MR-1 after expression from the corresponding gene in a pBAD202/D-TOPO vector (see ESI Section 1† for more details). We have previously shown that the *Ser*87 to *Cys* mutation has no effect on the overall STC secondary and tertiary structure.^35^ The STC's *Cys*87 residue was used to covalently link STC to a freshly cleaned Au(111) substrate by exposing a clean Au surface to a 20 μM solution of protein in 20 mM Tris buffer (pH 7.5), 100 mM NaCl aqueous solution for 30 min, followed by washing with Milli Q water. The STC-modified Au electrode was then mounted onto the STM cell and the cell filled with water or 0.1 mM Hepes buffer (pH = 7). A freshly electrochemically etched Au tip was insulated with Apiezon wax and used as the STM tip electrode. Individual adsorbed proteins can be resolved in STM images as bright spots (Fig. 2a). Note that the apparent height of proteins in STM always appears much lower (typically <1 nm for a protein of 3–4 nm^36^) due to the conductivity difference between the metal Au substrate and the protein. To avoid formation of multi-protein junctions during the single-protein transport measurements, the protein surface coverage was always kept to below a full protein monolayer (see ESI Fig. S10 and S11†). Low protein coverage on the electrode surface also enables the gap distance calibration through the electrode–electrode empty gap (see ESI Fig. S4†). The successful STC/electrode covalent attachment was evidenced by the stable STM images upon successive scans similar to previously studied redox protein systems.^24,37–40^ The lack of the surface *Cys*87 resulted in blurry, unstable protein imaging (see STM images for a wild-type (unmodified) STC, ESI Fig. S11†) evidencing surface protein anchoring proceeds *via* strong Au–S(*Cys*) covalent chemistry.

### Dynamic single-protein charge transport measurements

The dynamic STM-break junction (STM-BJ) approach has been extensively described.^41,42^ Briefly, the STM tip is first brought to a tunnelling distance over an atomically flat Au(111) substrate functionalized with STC (Fig. 2a). The STM feedback is then turned off and the tip driven in and out of contact to and from the Au substrate at 20–40 nm s^−1^. The STM tip is retracted several nanometres beyond the position where a zero current is detected (“out of contact” position) where no protein is trapped between the two electrodes and the tunnelling gap is empty. Then, it is approached back again towards the Au surface until the current almost reaches saturation (“in contact” position). This two-points feedback loop is used to collect thousands of current decays, *G*(*z*), (∼3000) during the tip pulling cycles (Fig. 2b inset), being *G* the conductance of the nanoscale junction and *z* the electrode–electrode gap separation. To minimize the damage to the STM tip and sample surface, the maximum current of the two-points feedback loop was set to a value just below current saturation, which prevents vigorous crashing of the STM tip onto the substrate electrode. The appearance of clean steps (plateaus) in the *G*(*z*) in the dynamic STM-BJ experiments (Fig. 2b inset) denotes formation of stable single-molecule bridges between the Au surface and the STM tip. The current level at which the plateau appears is then used to determine the single-molecule conductance using the expression *G* = *I*_step_/*U*_bias_ where *G* is the conductance, *I* is the current and *U* is the potential difference between the two junction electrodes. An automated selection procedure designed on LabVIEW was used to separate the current decays that showed plateaus from the ones that did not. High experimental yields of *G*(*z*) showing plateaus were typically obtained in the experiments ranging between 40 and 60% of the total collected traces (1200–1800 traces), which were then used to build conductance histograms. Fig. 2b (see also ESI Fig. S1†) shows a semi-logarithmic conductance histogram of all selected traces displaying current plateaus. The observed peaks in the conductance histograms correspond to the probabilistic appearance of a plateau at a particular conductance *G* in the *G*(*z*) trace and provide an average of the single-molecule conductance of all stable, plausible molecular junction configurations. A visual inspection of the individual traces show the appearance of conductance plateaus distributed in four different broad *G* regions (Fig. 2b coloured bands). These different *G* regions arise in the histograms as four deconvoluted peaks (see Fig. 2b and ESI Fig. S1†) using Gaussian distribution functions yielding average conductance values for each of the 4 different regions: (1.5 ± 2.5) × 10^−5^*G*_0_, (1.5 ± 2.0) × 10^−4^*G*_0_, (5 ± 1.2) × 10^−4^*G*_0_ and (1.62 ± 1.5) × 10^−3^*G*_0_ (where *G*_0_ = 2*e*^2^/*h* ≈ 77.4 μS). The error intervals in conductance values were extracted from the FWHM of the fitted Gaussian peaks in the 1D histograms (Fig. 2b). To check the consistency of the dynamic transport data, measurements were conducted at different applied bias voltages of 20, 50, 100, 150 and 200 mV (ESI Fig. S1†) yielding similar peak distribution in all cases.

The observed multiple plateaus in the individual *G*(*z*) traces as a function of increasing electrode–electrode gap separation indicate specific interactions of the junction electrodes with different points of the protein surface, *i.e.* particular electrode/protein configurations in the tunnelling junction,^43,44^ leading to distinct electron pathways being probed along the STC backbone. Note that the longest pulling distances above 2 nm recorded for the low conductance features (left trace in Fig. 2b) indicates a partial lifting of the protein which always stays at a high tilt angle from the electrode surface upon junction breakdown (Fig. 2c).

### Static single-protein charge transport measurements

**Fig. 3:**
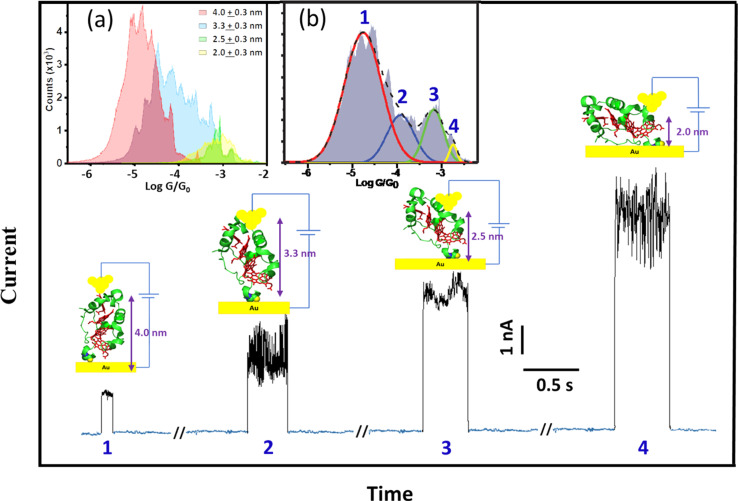
Representative *G*(*t*) blinking traces with subtracted background tunnelling current obtained at different electrode–electrode separations of the STM nanoscale gap. Insets: (a) conductance histograms accumulating blinks at the different electrode–electrode gap separations of 2.0 ± 0.3, 2.5 ± 0.3, 3.3 ± 0.3 and 4.0 ± 0.3 nm sorted by colours (see graph legend) represented in a common graph (error bars in the gap separations are calculated from the error in the experimental beta decay determination through the empty junction (see Fig. S4†)). (b) Deconvolution of the cumulative histogram of all 1686 traces without gap distance classification. The applied bias voltage was 200 mV.

To support the dynamic STM-BJ results, we performed static STM-BJ experiments under controlled fixed electrode–electrode gap separations. This method allows charge transport measurements under a defined tunnelling gap size. Briefly, in a static STM-BJ experiment, the STM gap is mechanically and thermally stabilized at a pre-set tunnelling current. Then, the tunnelling current feedback is switched off and current transients, *G*(*t*), at the fixed electrode–electrode gap separations are captured. The gap size was set by approaching the STM tip to the Au substrate until a low tunnelling setpoint current flowing between the two electrodes is reached, typically from few hundreds of fA to few tens of pA, depending on the proficiency of the STM tip electrical insulation. The experimental quantification of the current decay through the empty gap measured on the same semi-covered protein layer allows estimating the gap separation for a set tunnelling current (see electrode–electrode gap calibration in ESI Fig. S4†). After that, the tip is further separated to a pre-set distance using the calibrated STM piezoelectric scanner to reach the desired final electrode–electrode gap size. When a protein spontaneously spans the gap between the two electrodes, an abrupt jump in the transient current is observed in the form of a telegraphic signal (Fig. 3).^42,45^ We refer to these current jumps as “blinks” and this type of experiments as blinking experiments. A blink typically last for a short period of time (from tens to hundreds of ms), after which the current suddenly drops to the initial background level due to the spontaneous breakdown of the molecular junction. In order to ensure that the recorded blinks are due solely to the formation of molecular junctions, a mechanically induced STM tip pulling^46^ is applied during the lifetime of the blinks (ESI Fig. S2b†). The appearance of plateau-like features as the junction is forced to break *via* the mechanical pulling evidences that the blink was caused by the formation of a molecular bridge. Unlike the dynamic STM-BJ approach, which forces the tip in and out of the surface and therefore may lead to stretch-dependent conductance of the protein, the spontaneous formation of junctions in blinking experiments can be envisioned as a method to momentarily stabilize particular conformations of an individual molecule in the tunnelling gap.^14,47,48^ The average blinking lifetime provides information about the strength of the electrode/molecule interaction for every particular protein orientation in the nanoscale junction. Conductance histograms (Fig. 3) and 2D maps (Fig. S3†) are built by the accumulation of 1500+ individual blinks with no data selection. We collected *G*(*t*) traces on STC at various electrode–electrode gap separations; 2.0 ± 0.3, 2.5 ± 0.3, 3.3 ± 0.3 and 4.0 ± 0.3 nm. Fig. 3 shows illustrative static *G*(*t*)s showing blinks for each of the measured electrode–electrode gap separation. An inverse dependence of STC conductance on the gap separation is observed, in contrast to what we observed in previous work with other redox proteins, where gap separations did not affect protein conductance (ESI Fig. S10a†).^24^Fig. 3a inset represents the individual histograms obtained at each gap separation (see colour legend) accumulating all *G*(*t*) blink traces with no data selection. Note that at the two shorter gap separations, 2 and 2.5 nm (differing by a shorter 0.5 nm distance), the tunnelling gap is not able to discern on average between the two different configurations of the protein in the gap leading to the two highest *G* values appearing indistinctly in both gaps (see also ESI Fig. S3†). However, when all *G*(*t*) blinking traces (1686 in total) are accumulated in a single histogram without gap distance sorting (Fig. 3b inset), an equivalent conductance peaks distribution compared to the one obtained in the dynamic experiments arises from the peak deconvolution analysis. Four distinct conductance features show up at (1.8 ± 1.5) × 10^−5^*G*_0_ and (1.2 ± 3.0) × 10^−4^*G*_0_, ascribed to the 4.0 ± 0.3 nm and 3.3 ± 0.3 nm gap separations respectively, and at (6.7 ± 3.1) × 10^−4^*G*_0_ and (1.8 ± 1.3) × 10^−3^*G*_0_ both observed indistinguishably at the 2.0 and 2.5 nm gap separations (labelled 1–4 in Fig. 3b). Error bars in the conductance values are calculated from the FWHM of the Gaussian fits of the histogram (Fig. 3b inset). A few observations are worth highlighting here: (1) the good conductance agreement between the blinks obtained at the large gap separations (4 nm) in the static experiment (labelled as 1 in Fig. 3a and inset 3b) and the low conductance plateaus in the dynamic experiment (Fig. 2b) suggests that the conductance along the entire long protein axis is being probed in both cases. (2) The longer lifetimes observed for the fully extended configuration, *i.e.* longer blink's lifetimes and step lengths in static and dynamic modes respectively, suggest a stronger protein/electrode interaction in this case. And (3), the good general correspondence between the dynamic and static charge transport data supports the STC molecules being structurally robust against the STM-BJ such that the protein does not significantly deform during the pulling stage of the dynamic STM-BJ method.

### Step length analysis

**Fig. 4:**
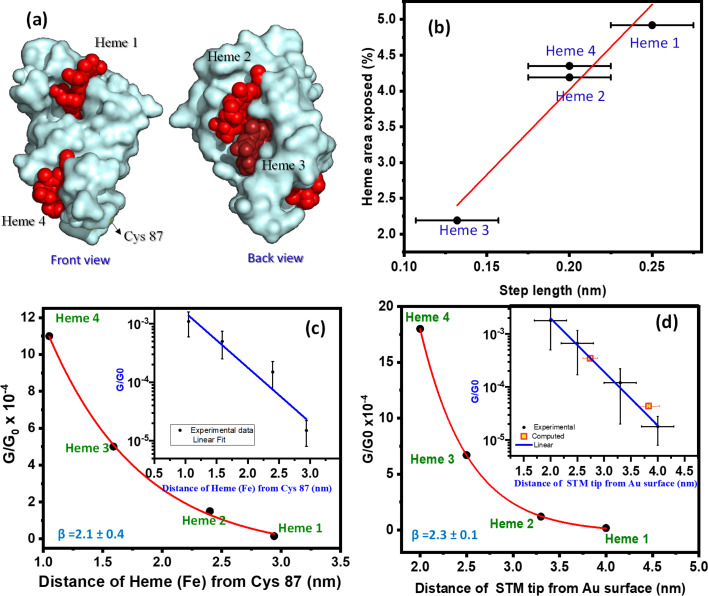
(a) Solvent accessible surface area (SASA) representation of the area of exposed *hemes* 1–4 (in red). (b) Representation of step length calculated from dynamic STM-BJ *G*(*z*) plots *versus* the percentage of exposed *heme* area calculated using SASA of the protein crystal structure (1m1b pymol). (c) Conductance decay plot representing the STC conductance values extracted from the dynamic STM-BJ histograms against the protein crystallographic distances (Fig. 1) from the bottom contact point (*Cys*87) to each *heme* 1–4 (calculated using pymol 1m1q). Inset: semi-log representation of *G*/*G*_0_*versus Cys*87-to-*heme* distance, fitted to a straight line (blue) yielding *β* = 2.1 ± 0.4 nm^−1^. (d) Conductance decay plot representing STC conductance extracted from the static STM-BJ histograms against the electrode–electrode junction gap separation. Inset: semi-log representation of *G*/*G*_0_*versus* the electrode–electrode gap separation, fitting a straight line (blue) which yields a decay constant of *b* = 2.3 ± 0.1 nm^−1^. The two filled (yellow) squares are computed DFT conductance values for two averaged standing and lying junction configurations (see ESI Fig. S6†). All error bars in conductance values were extracted from the FWHM of the fitted Gaussian peaks in histograms. Error bars in the electrode–electrode gap separations were extracted from the error in the experimental *β* determination through the empty gap (see calibration in ESI Fig. S4†).

We explore the surface chemistry of the STC crystal structure (Fig. 1) to identify chemical groups involved in possible specific protein/electrodes interactions that could lead to the observed four different conductance configurations of the STC in the tunnelling junction. Similar to procedures used by us and others to immobilized globular proteins on a metal surface,^22,24,25,39^ the surface *Cys*-modified STC at the *Ser*87 position is used to covalently link STC to a freshly prepared atomically flat Au surface *via* the thiol group of the surface exposed *Cys* (Fig. 1 and 2c). This allows the formation of a strong covalent bond between the protein and the substrate STM electrode as evidenced by the STM imaging of a wild-type (unmodified) STC (ESI Fig. S11†). Moreover, the *heme* redox cofactors are highly exposed to the electrolyte and their side groups extend out of the protein structure. We and others^27,29,49,50^ have extensively studied charge transport behaviour in a number of *heme* homologous metalloporphyrins and demonstrated that the large affinity between a Au surface and the π-system of the porphyrin ring yields stable molecular junctions. The latter made us consider the exposed *heme* co-factors as the possible connecting points between the STC protein and the STM tip electrode. *Hemes* are arranged in a ladder like fashion following a TST array (Fig. 1) with very distinct solvent exposure among the different *hemes* 1–4 (Fig. 4a). We then look for a correlation between the solvent-exposed area of each individual *heme* co-factor and the step length of the current plateaus obtained in the dynamic STM-BJ experiment, which represents the maximum pulling distance before the relevant electrode/protein interaction breaks. The step length in a dynamic STM-BJ experiment scales with the interaction strength between the molecule and the junction electrodes^51^ and, therefore, a larger Au electrode overlap with a more solvent-exposed *heme* porphyrin ring is expected to result in a larger protein/electrode interaction, which will result in a longer dynamic plateau length (Fig. 2b inset). The average plateau lengths are extracted from the Gaussian-fitted peaks of the current *versus* electrode–electrode separation histograms accumulating all dynamic *G*(*z*) current traces (ESI Fig. S5†). The results are summarized in ESI Table 1.† To calculate most accessible *heme* surface areas, we have used solvent-accessible-surface-area (SASA) calculations using the crystal structure of the protein. Out of the total protein area, 5956.85 Å^2^, the area for each *heme* was found to be *heme*-4: 259.34 Å^2^ (4.35%); *heme*-3: 130.65 Å^2^ (2.19%); *heme*-2: 249.66 Å^2^ (4.19%); *heme*-1: 293.48 Å^2^ (4.92%). We found a linear correlation between the observed plateau length and the % of exposed *heme*, *i.e.*, the plateau length increases with the increasing *heme* exposed area (Fig. 4b). Note that the close SASA values between *hemes* 2 and 4 lead to indistinguishable plateau lengths. Overall, this correlation suggests that as a larger area of the porphyrin π-system is readily available for interaction with the metal electrode, a stronger electrode/*heme* interaction is established which translate into longer plateau length in the dynamic STM-BJ experiment.

Since we found good statistical correlation of both (1) number of observed conductance plateaus *versus* number of *heme* cofactors, and (2), step length of conductance plateaus *versus heme* SASA values, we suggest that the different conductance states represent STM tip/*heme* (1–4) specific interactions probing different number of *heme* cofactors in the electron pathway. The latter picture is also supported by the increasing number of conductance states as more *heme* cofactors are present in the protein structure (see dynamic transport data for a longer decaheme MHC, ESI Fig. S10b†) and by the lack of multiple conductance states in a globular Cu-redox protein (ESI Fig. S10a†). It is also worth highlighting the agreement between the larger average plateau length observed for the most exposed *heme* 1 in the dynamic charge transport experiments (ESI Table 1†) and the average longer lifetimes recorded for the larger electrode–electrode gap separations (Fig. S3†), where the STM electrode is also expected to interact with the most solvent-exposed and distal *heme* 1.

The above results allow for a direct estimation of the electron decay constant along a STC wire. The conductance values corresponding to electron pathways involving 1 to 4 *heme* cofactors are plotted against the relevant tunnelling distance calculated in two different ways: (1) as the protein crystallographic distance (Fig. 1) between the corresponding *heme* and the bottom Au/*Cys*87 contact point for the dynamic experiments, and as the estimated electrode–electrode gap separation for the static experiments (Fig. 4c and d, respectively). The plots show clear exponential decays in both cases yielding similar decay *β* factors of 2.1 ± 0.4 nm^−1^ and 2.3 ± 0.1 nm^−1^ for the dynamic and static STM-BJ experiments, respectively.

### Current *vs.* voltage characteristics & computational simulation

The results obtained from both static and dynamic single-protein charge transport data are consistent with the pictorial representations of the protein junctions shown in Fig. 2c and 3, where electron pathways involving a different number of *hemes* from 1 to 4 are probed in the single-protein wire.

**Fig. 5:**
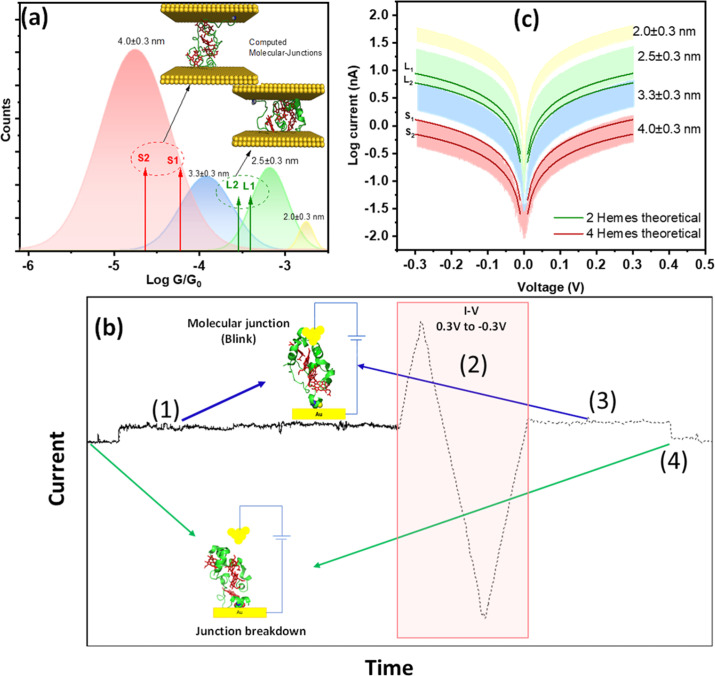
(a) Deconvoluted Gaussian functions extracted from Fig. 3b inset represented in different colours as per each identified gap separation against the computed conductance values from the different simulated structural configurations represented as red and green arrows, respectively, for standing STCs (4 *hemes* probed in the junction) S1(ox) & S2(ox) and lying STCs (2 *hemes* probed in the junction) L1(ox) & L2(ox). The S1 and L1 computed structures are included in the inset. (b) Representation of a successful *I*–*V* collection on a blink fulfilling the experimental sequence (1–4): (1) blink recognition at the selected electrode–electrode gap separation, (2) triggered voltage ramp on the blink, (3) identifying the original blink conductance level is recovered, and (4), forced pulling curve to identify protein junction breakdown. Inset: pictorial representation of open and closed junctions. (c) *I*–*V* characteristics measured at different electrode–electrode gap separations of 4.0 ± 0.3, 3.3 ± 0.3 and 2.5 ± 0.3 and 2.0 ± 0.3 nm, shown as bands representing the total current dispersion per each gap distance. Overlayed solid lines represent computed *I*–*V* curves of STC junctions using DFT, where red lines represent S1(ox) & S2(ox) and green lines represent L1(ox)& L2(ox). Error bars in the electrode–electrode gap separations were extracted from the error in the experimental determination of *β* through the empty gap (see calibration in ESI Fig. S4†).

To support the above picture, we generated atomistic models of solvated STC junctions and computed their current–voltage response using density-functional-theory (DFT) based electronic structure calculations (see Fig. 5a and ESI Fig. S6†). *Heme*-to-*heme* electron hopping with full charge relaxation is ruled out in the present measurements because the measured currents in the formed single-protein wires are more than two orders of magnitude faster than the fastest *heme*-to-*heme* electron transfer rate for solvated STC as predicted by computation^52^ and then independently confirmed by pump-probe transient absorption spectroscopy.^53^ Thus, we use a coherent transport model in the calculations. The generated junction models represent two ‘lying’ STC structures (L1 and L2), where the transport is mediated by two *heme* cofactors, and two ‘standing’ structures (S1 and S2), where the transport pathways involve all four *hemes* (Fig. 5a). Electrode–electrode separation distances in these models are 2.68, 2.81, 3.63, and 4.05 nm, respectively, spanning a similar range as in experiments (ESI Fig. S6†). The theoretical electron tunnelling decay factor extracted from the two computed configurations yields *β* = 1.8 ± 0.2 nm^−1^, which is in very good agreement with the experimental measurements discussed above. Also, the calculated mean conductance values of ‘lying’ and ‘standing’ structures in their oxidised form, (3.5 ± 0.2) × 10^−4^*G*_0_ and (4.35 ± 0.2) × 10^−5^*G*_0_, respectively, where the transport is mediated by 2 and 4 *hemes*, respectively (ESI Fig. S8†) correlate well with the corresponding experimental results (see Fig. 4d inset and 5a). Whilst we were unable to generate model structures where transport is mediated by 1 or 3 *hemes*, the good agreement between theory and experiment for the 2 and 4 *heme* structures strongly supports the protein junction pictures represented in Fig. 2c and 3.

The above comparison between experiments and simulation is complemented by recording experimental current *vs.* voltage (*I*–*V*) characteristics of the single STC junctions at the different electrode–electrode gap separations, which will enable direct comparison to the calculated transmission functions. The *I*–*V* analysis does not bring new insights beyond the conductance comparison presented in Fig. 5a and they cannot be done at the same statistical level as compared to the above dynamic and static STM-BJ experiment. It however serves as an additional point of comparison between simulations and experiments of individual STC conductance, enabling direct fitting of the experimental data to the calculated transmission curve. To ensure the included *I*–*V*s in the experimental averages are those coming exclusively from stable single-STC junction, we designed the experimental sequence sketched in Fig. 5b: (1) a blink matching the statistical conductance observed in Fig. 3 for the relevant gap separation is detected indicating protein junction formation. (2) A fast scan (0.1 s) cyclic *I*–*V* of ±0.3 V is triggered during the blink lifetime. (3) After the *I*–*V* is completed, the conductance remaining at the same initial level indicates the protein junction survived the voltage ramp. (4) Finally, to ensure the *I*–*V* was collected on a single-protein junction, the electrode–electrode gap is opened, and the decay current simultaneously collected (Fig. S2b†). The appearance of step features in the latter indicates the presence of a protein trapped in the junction (Fig. S2b,† left panel), as opposed to a false empty junction reading, which would yield a clean exponential decay (Fig. S2b,† right panel). Due to the inherent instability of the single-protein junctions, out of 500+ collected *I*–*V*s at each gap separation, few tens only (40–60 curves) fulfilled all the steps of the experimental sequence described above (Fig. 5b) and were added to the *I*–*V* representations in Fig. 5c (and ESI Fig. S8†). The bands in Fig. 5c show the full dispersion of *I*–*V*s where each colour represents each probed electrode–electrode gap separation. The *I*–*V*s recorded at the same four electrode–electrode gaps used in the above static STM-BJ experiments showed similar four regions distribution of conductance values. The results for each electrode–electrode gap separation were averaged to get the average conductance and standard deviation which was represented as error bars in Fig. S8b.† DFT computed *I*–*V* curves of STC in the standing (4 *hemes*) and lying (2 *hemes*) configurations (solid lines in Fig. 5c and ESI Fig. S8c†) both correlate well with the experimental data.

## Conclusion

Multiheme proteins are the building blocks of bacterial transmembrane and extracellular structures displaying charge transport capabilities within exceedingly long length scales in the micrometre range.^8,9^ Despite the general structural information available for such systems, there is still an open debate about the origin of their outstanding charge transport properties.^54–57^ The structural ingredients leading to long-range charge transport in such biological structures would lead us to the biomimetic design of a new generation of conductive organic polymers for their implementation in energy efficient hybrid bioelectronics. In this work, we have measured for the first time the conductance and electron tunnelling decay constant of a single small tetraheme protein (STC) from *Shewanella oneidensis* in an aqueous environment. STC allows for the study of charge transport in an isolated TST array composed of four *heme* cofactors, which is the repeating structural unit found in large MHC complexes forming part of the conducting bacterial nanostructures. By trapping STC proteins in a dynamically controlled tunnelling gap immersed in the aqueous electrolyte, we record four distinct protein conductance values spanning from 10^−3^*G*_0_ to 10^−5^*G*_0_ when the probed electron pathway involves one to four *heme* cofactors, respectively. We rely on three key facts to support the above picture: (1) the number of statistically observed conductance features (peaks) at increasingly larger gap separations of the nanoscale tunnelling junction matches the number of solvent-exposed *heme* co-factors, (2) the formation of stable metal/porphyrin junctions has been extensively reported^58^ and there is absence of other solvent-exposed chemical groups at the protein surface prone to form stronger chemical interactions with the metal STM tip, (3) the experimental correlation between the plateau length in the dynamic experiments and the percentage of solvent-exposed area of each *heme* co-factor is a strong evidence of the STM tip/*heme* binding occurrence, and (4), there is absence of multiple conductance states as a function of the tunnelling gap separation in previous single-protein junction determinations studying non-*heme* globular proteins.^24,33^ The agreement of the experimental conductance values with the computed electric currents for a single-STC protein trapped between two electrodes suggests the TST moiety is able to support efficient electron tunnelling as the mechanism for charge transport. The low recorded beta decay along the STC wire, ∼2.2 nm^−1^, is similar to that of a conjugated molecular wire of similar length such as polyfluorene wires,^59^ which suggest low tunnelling energy barriers. Previous measurements^25^ and computations^57^ have suggested that the coherent tunnelling regime extends to even larger MHC proteins. Clearly, at some distance, coherent tunnelling across the junction will become uncompetitive due to its exponential distance dependence and will give way to incoherent or mixed coherent-incoherent mechanisms. The results suggest coherent-assisted electron diffusion could be a plausible scenario, where individual TST motifs might act as independent “stepping stones” units in a hopping-coherent mixed mechanism across long MHC-based structures. Such mechanisms has been proposed as a way to generate superior electron diffusion in long-range conduction through a long (hundreds of nm) MHC chain.^34^ The results and new methodologies developed in this work also bring a new fundamental platform to in-detail study mechanisms of charge transport in MHC proteins by combining single-protein electronic approaches and mutagenesis. This will open to understanding the observed long-range charge transport in MHC-based structures exploited in several type of bacteria.

## Data availability

All relevant data are presented in the paper and ESI.† Raw data are available upon request by email to the corresponding author.

## Author contributions

I. D.-P., J. B. and J. N. B. acquired funding, conceptualized the project and designed the methodology. K. G., Z. F., X. W., Y. J. and V. C. P. performed experiments and analysis and polished the methodology. J. H. W. and R. C. contributed to the methodology and sample preparation and conducted protein production and purification. T. Q. H. and A. C. A. supervised research and conducted data analysis. K. G., I. D.-P., J. B. and J. N. B. all contributed to write and edit the manuscript.

## Conflicts of interest

The authors declare no competing financial interest.

## Supplementary Material

SC-015-D4SC01366B-s001
