# Supplementary material for: Shallow conductance decay along the heme array of a single tetraheme protein wire
Source: Chem Sci. 2024 Jul 3;15(31):12326–35. doi: 10.1039/d4sc01366b (PMC11304805; doi:10.1039/d4sc01366b)
Supplement: SC-015-D4SC01366B-s001 [file SC-015-D4SC01366B-s001.pdf]

Electronic Supplementary Material (ESI) for Chemical Science.
This journal is © The Royal Society of Chemistry 2024

Electronic Supplementary Information (ESI)

Shallow conductance decay along the *Heme* array of a single Tetra*heme* protein wire

Kavita Garg,[1] Zdenek Futera,[4] Xiaojing Wu,[2] Yongchan Jeong,[1] Rachel Chiu,[3] Varun Chittari Pisharam,[1] Tracy Ha,[1] Albert C. Aragonès,[5] Jessica H. van Wonderen,[3] Julea N. Butt,[3,*] Jochen Blumberger,[2,*] Ismael Díez-Pérez[1,*]

[1] *Department of Chemistry, Faculty of Natural, Mathematical & Engineering Sciences, King's College London, Britannia House, 7 Trinity Street, London SE1 1DB, UK.*

[2] *Department of Physics and Astronomy and Thomas Young Centre, University College London, Gower Street, London WC1E 6BT, UK.*

[3] *School of Chemistry and School of Biological Sciences, University of East Anglia, Norwich Research Park, Norwich, NR4 7TJ, UK.*

[4] *Faculty of Science, University of South Bohemia, Branisovska 1760, 370 05 Ceske Budejovice, Czech Republic.*

[5] *Departament de Ciència de Materials i Química Física, Universitat de Barcelona and Institut de Química Teòrica i Computacional (IQTC), Marti i Franquès 1, 08028 Barcelona, Spain.*

* Corresponding address: J.Butt@uea.ac.uk, j.blumberger@ucl.ac.uk, ismael.diez_perez@kcl.ac.uk

1. Methodologies, Dynamic charge transport at different bias voltages, control experiments, static charge transport 2D maps, step length histograms and SASA analysis

STC and MtrC production. The S87C STC protein was purified from *Shewanella oneidensis* MR-1 after expression from the corresponding gene in a pBAD202/D-TOPO vector. An N-terminal Strep II-tag was introduced to facilitate protein purification. Full details of protein preparation for both S87C and wild-type (unmodified) STC are provided elsewhere.[1] Protein purity was confirmed by SDS-PAGE (Fig. S9). LC-MS analysis reveals a single-peak corresponding to a mass of 13561 Da in excellent agreement with that predicted (13558 Da) for the mature protein with four covalently bound *Hemes*. Aliquots of purified S87C STC (200 μM) in 20 mM TRIS, 100 mM NaCl, pH 8.5 were stored frozen at 193 K. CD spectrum of the protein in solution was measured to check the protein secondary structure. A similar protocol was followed for the preparation of the MtrC with full details given elsewhere.[23]

STM sample preparation. Au (111) crystals were cleaned using Piranha solution (7:3 H2SO4/H2O2 (30%) by volume), (Caution: *Piranha solution should be handled with extreme caution*) followed by H_2 flame annealing to prepare atomically flat gold surfaces. To attach STC on gold through Ser87 mutated to *Cys*, freshly annealed and cooled crystal was incubated with solution of the S87C STC variant (20 μM) in 20 mM TRIS, 100 mM NaCl, pH 8.5 which results in a defined orientation of the protein on the surface.[2]

STM experiment. Deionized water (18 MΩ cm−1 Milli-Q, Millipore) was used for rinsing samples and electrodes. ECSTM probes were prepared by electrochemical etching of a 0.025 mm diameter Au wire (99.99%), briefly flame annealed and isolated with Apiezon wax. Single-molecule experiments were performed with a PicoSPM microscope head and a PicoStat bipotentiostat (Molecular Imaging) controlled by a 2100 Agilent SPM control unit. The data was acquired using NI-DAQmx and BNC-2110 LabVIEW acquisition system and analysed with LabVIEW and Origin programs.

Dynamic STM-BJ experiments have been describe in the main manuscript and extensively elsewhere.[3,4] To build the conductance histograms, the same homemade LabVIEW algorithm was used to identify plateau features in the dynamic pulling traces, which were observed with a high yield in 40-60% of the collected traces. Plateau-bearing dynamic traces were then selected and directly accumulated to build histograms. This procedure allows rejection of highly noise and featureless traces (see Fig. S2d) which substantially improves data quality.[4,5]

Static STM-BJ transient recordings were collected to analyse the spontaneous formation of single-protein wires. In this case, after bringing the probe to a tunnelling distance from the substrate, the STM feedback was turned off and the current was recorded as a function of time. When a molecule bridges between both junction electrodes, a sudden "jump" or "blink" in the current is detected (I_{blink}).[5] To confirm that an individual STC was bridging the two junction electrodes, the molecular bridge was eventually broken intentionally by pulling one of the junction electrodes away from the surface using the piezoelectric actuator, as reported before in the literature. The magnitude of the blink can be used to calculate the single-protein conductance using $G = I_{blink} / U_{bias}$. Probability histograms were obtained by subtracting the initial current setpoint from the current–time raw data trace and binning the traces afterwards. Since the blinking process is completely stochastic, all blinking trace were then identified and cut with an automated homemade LabVIEW/MATLAB algorithm and set to a common time

zero. The latter blinking lines were then all accumulated into the 1D and 2D histograms without any further selection. Cumulative histograms were obtained by the same method from random groups of traces under the same experimental conditions to test the heterogeneity of the populations.

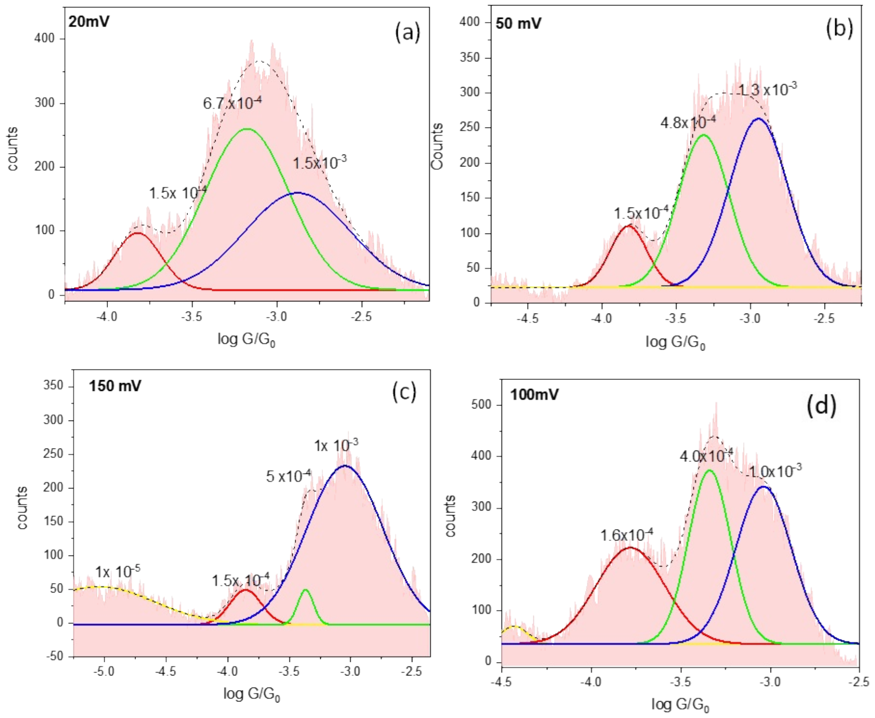

Figure S1. *(a)-(d): Low resolution dynamic STM-BJ (~500 traces) semi-log 1-D histogram at 20mV, 50 mV, 100mV, and 150mV. Deconvoluted Gaussian fits of the individual peaks are used to extract the maxima conductance values. Note that the low conductance feature is only resolved at bias voltage >100 mV due to the low currents associated with this state.*

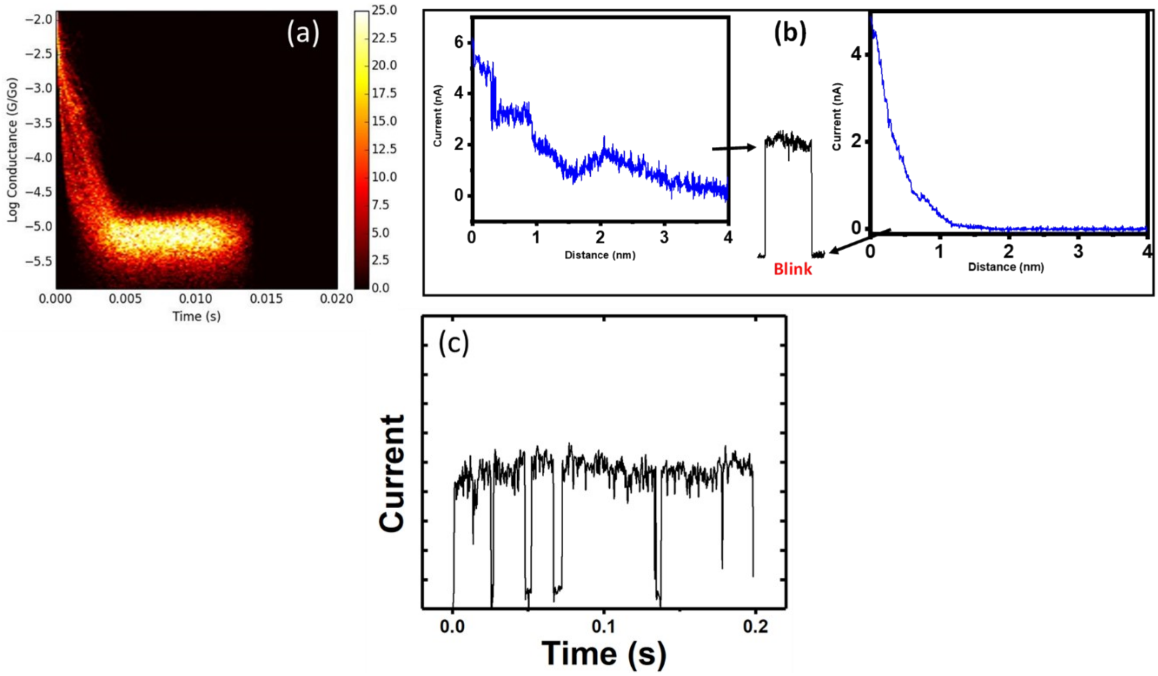

Figure S2: *(a) Control 2D heat map for a dynamic STM-BJ experiment on clean Au in HEPES buffer showing a clean exponential decay. (b) Schematic representation of junction pullings done at a blink feature to confirm the presence of molecule; Left panel represents the pulling curve obtained at the blink, where steps-like features suggest the presence of a molecular junction; Right panel represents a pulling curve obtained at the baseline, where a clean (no plateau features) exponential decay suggests that the tunnelling gap is empty. (c) Individual "blink" trace for a typical static experiment showing breaking down and reformation of a protein junction. The junction current is recovered back to the same level in every breakdown/formation event.*

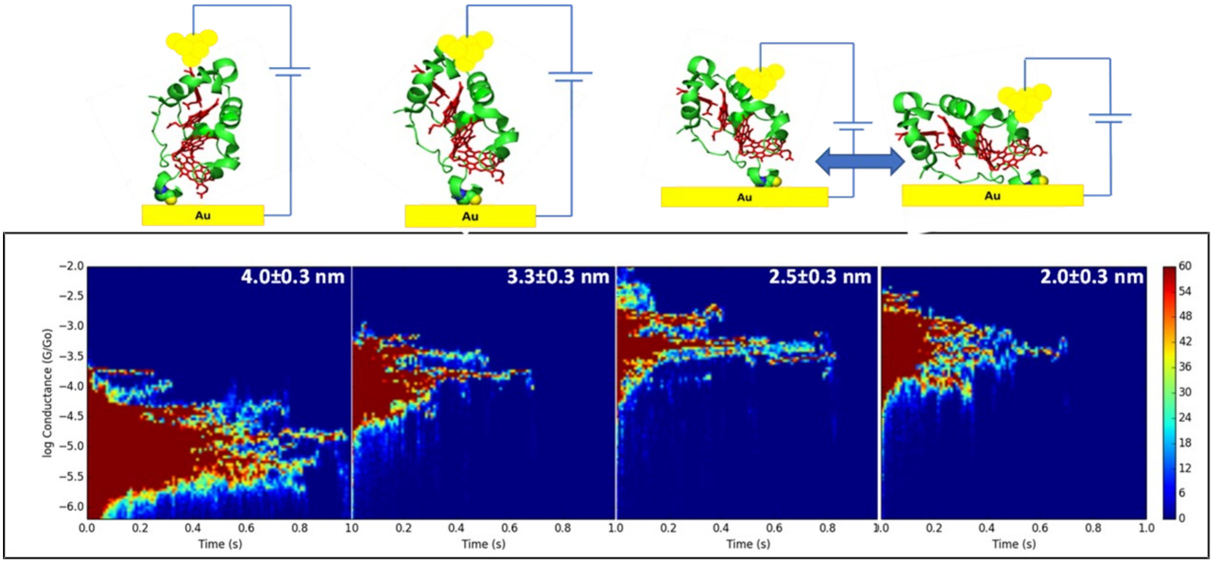

Figure S3: *2D histograms accumulating "blinking" current transients in various static STM-BJ experiments conducted at different tunnelling gap separations (electrode-electrode distances) of 2.0±0.3 (178 traces), 2.5±0.3 (210 traces), 3.3±0.3 (210) and 4.0±0.3 nm (750*

traces). All blink features are cut and placed in a common time origin, and the tunnelling baseline subtracted. The shortest 2.5 and 2 nm gap separations display both a similar conductance distribution suggesting they both can accommodate electron pathways including one or two heme groups (see cartoons in the top panels).

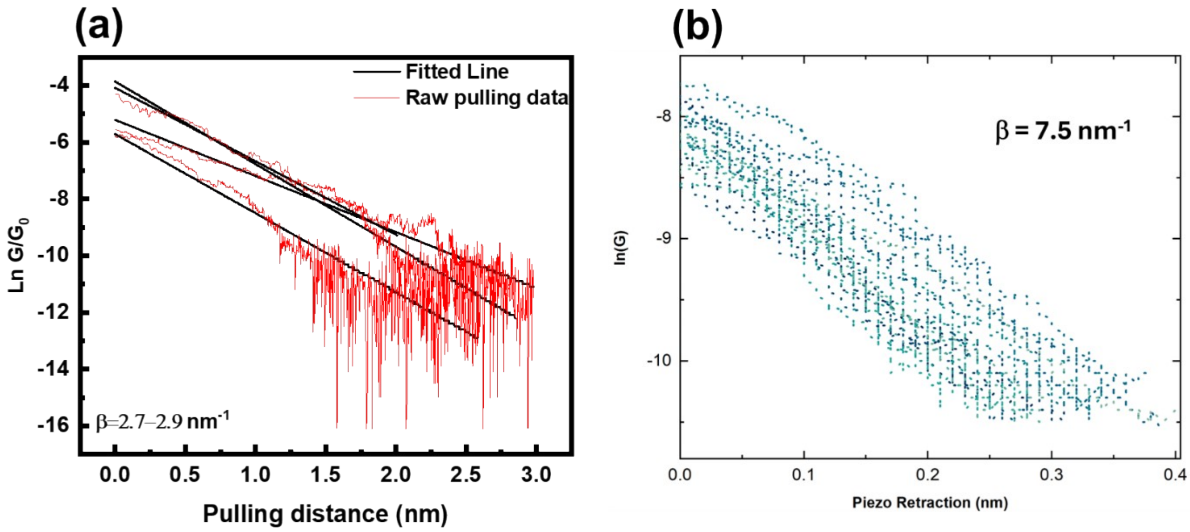

Figure S4: *Experimental determination of the electrode-electrode gap separatioon. (a) G(z) (LnG/Go vs. pulling distance (nm)) plots for empty junctions (no protein in the tunnelling junction) measured under the same experimental conditions used for the blinking experiment, i.e., 0.1 mM Hepes buffer, apiezon coated Au STM tip and STC-functionalized Au(111) surface under ambient conditions. Empty gaps display clean exponential decay traces (representative curves in the Fig. plot) from where an average decay constant through the tunnelling gap of* $\beta = 2.8 \pm 0.1\ nm^{-1}$ *is obtained using the relation* $Ln\ G/G_0 = -\beta L$*, being L the tunnelling gap distance. This* β *value is then used to estimate the gap distance L in the blinking experiments by interpolating the employed setpoint tunnelling current value used to approach both tunnelling electrodes. We employ low coverage protein-functionalised electrodes in the STM measurements which allow using the* β*-calibrated empty gap at the beginning of each experiment to set the exact initial gap separation in the static experiment before the current feedback is tunrned off. The error in the* β *value calculation determines the distance error between the Au tip and surface shown in Figure 3a. (b) Same G(z) plots as in (a) for empty junctions on a clean Au surface immersed in an aqueous solution. High decay constant values are commonly obtained thorugh a liquid gap. This also shows the drastic change in metal work function upon chemical functionalization with STC (see (a)) and the need for distance calibration under the same working conditions as in (a).*

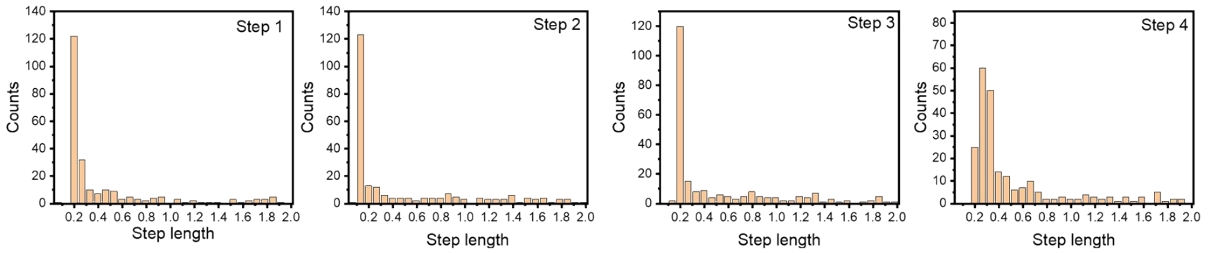

Figure S5: *Step length calculation for the G(z) plots displaying plateau features in the dynamic STM break-junction experiments at 100mV. Step 1 at G~$10^{-3}G_0$ (Heme 4: 1-heme electron pathway), Step 2 at G~$4x10^{-4}G_0$ (Heme 3: 2-heme electron pathway), Step 3 at G~$1x10^{-4}G_0$ (Heme 2: 3-heme electron pathway), Step 4 at G~$1x10^{-5}G_0$ (Heme 1: 4-heme electron pathway).*

Table S1. *Step lengths (calculated from the dynamic data) and percentage of exposed Heme area (calculated by SASA on the STC crystal structure, where the total surface area of STC is 5956.85 A2). SASA was obtained by the 'GETAREA' method using the web interface of the developers.[6]*

HEME	**Step Length (nm)**	**Area of *Heme* Exposed (Å^2)**	**% of *Heme* Area *exposed***
***Heme* 4**	0.20±0.025	259.34	4.35
***Heme* 3**	0.13±0.025	130.65	2.19
***Heme* 2**	0.20±0.025	249.66	4.19
***Heme* 1**	0.25±0.025	293.48	4.92

2. Atomistic model of solvated Au/STC/Au junctions

Solvated Au/STC/Au junction models were obtained by modification of the STC junctions generated in our recent study under vacuum conditions [7]. In that study, STC was docked in various orientations onto a gold substrate (bottom contact) followed by the adsorption of a second gold substrate (top contact). The STC structure was based on the X-ray structure from *Shewanella oneidensis* (PDB id 1M1Q) [8], mutated by replacing S87 by cysteine (S87C), and with N- and C-termini capped by acetyl and amine groups, respectively, in accord with the experimental setup in [9]. The junction contacts were modeled by two 6-monolayer (ML) gold (111) slabs of 6.153 x 6.090 nm^2 areas, to which the STC protein was adsorbed and covalently bound via Cys87 Au-S bonding to the bottom contact. The junction models were parametrized by GolP-CHARMM force field [12-14] based on CHARMM27 [15.16] and supplemented with

bis-histidine heme-cofactor and cysteine-linkages parameters, which we developed and used in our previous studies [7, 17-23]. Four model junctions, further denoted as **L1**, **L2**, **S1**, and **S2**, representing two “lying” (L) and two “standing” (S) adsorption structures with junction gold-to-gold widths 26.8 Å, 28.1 Å, 40.5 Å, and 36.3 Å, respectively, were prepared. The junctions in vacuum had zero net charges and neutral amino-acid side chains.

For the present simulation in an aqueous solution, we took the four junctions **L1**, **L2**, **S1**, and **S2** investigated in [7], solvated them with TIP3P water, and applied the standard amino-acid protonation states corresponding to pH 7. Protonation states of the heme-cofactor propionates groups were assigned based on their pK_a values predicted by PROPKA [10,11] (see Table S2 for details). Sodium counter-ions were added to neutralize the system. The solvated model was then padded by 10 Å thick vacuum layers below/above the bottom/top gold slab and treated in 3D periodic boundary conditions in the subsequent molecular dynamics (MD) simulations performed by Gromacs 2021.5 [25]. The solvated junction models were relaxed and equilibrated by 250 ns long MD performed with a 1 fs timestep. The electrostatic interactions were evaluated by the smooth particle mesh Ewald (PME) method [26,27] with a cutoff of 1.2 nm, while the Lennard-Jones short-range interactions were smoothly attenuated between 1.1 and 1.2 nm. Room temperature (300 K) was imposed on the system by the Nose-Hoover thermostat [28,29] during the simulations. Following the previous works [14,30], the GolP-CHARMM dipole temperature was set to 300 K, while the positions of the gold atoms were kept frozen. No constraints of bonds involving hydrogen atoms were applied. All four junctions remained stable during the MD runs, keeping their geometries (shown in Fig. S6) close to the ones described in [7] under vacuum conditions.

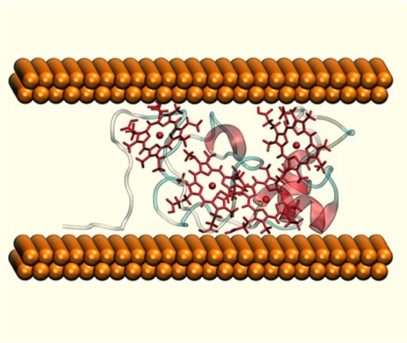
(a)

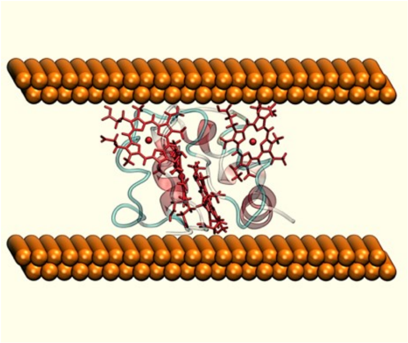
(b)

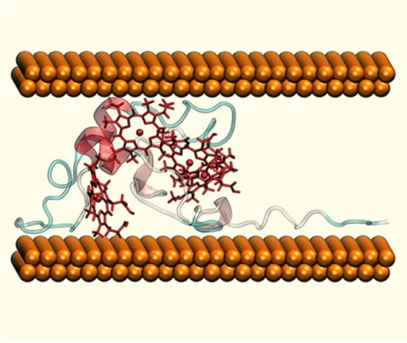
(c)

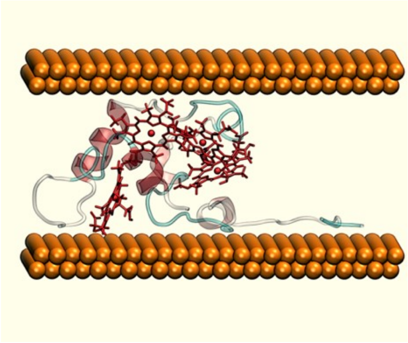
(d)

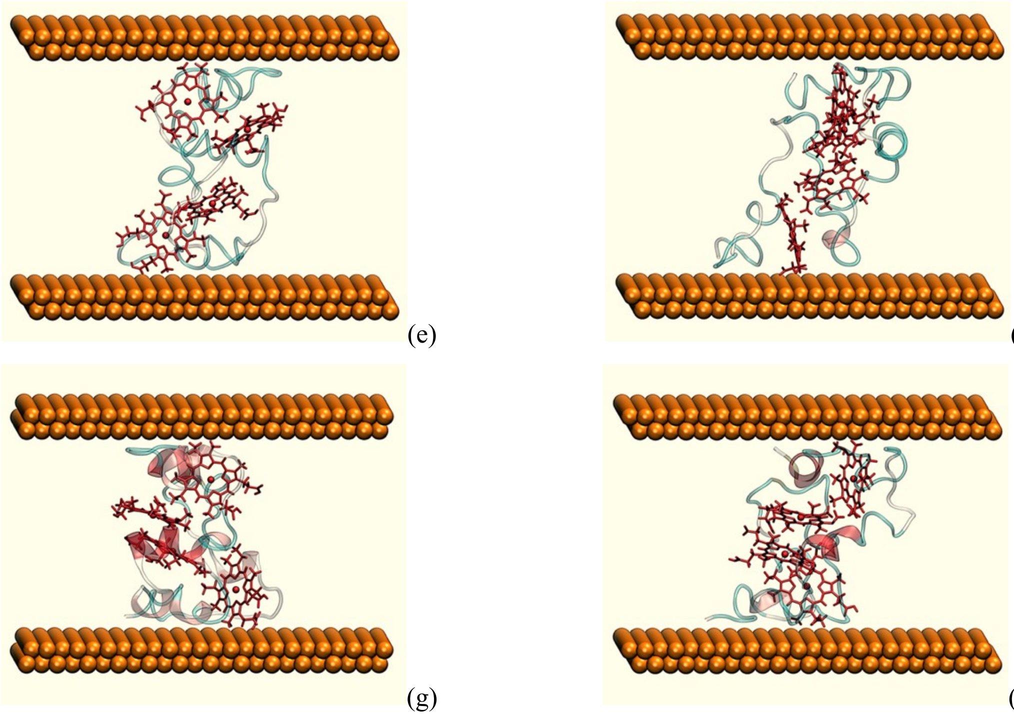

Figure S6: *STC junction models used for DFT calculations: (a) reduced **L1**, (b) oxidized **L1**, (c) reduced **L2**, (d) oxidized **L2**, (e) reduced **S1**, (f) oxidized **S1**, (g) reduced **S2**, (h) oxidized **S2**. Heme cofactors with Fe cations are shown in red, protein matrices are illustrated by transparent loops, and the gold electrodes are drawn by orange spheres.*

QM/MM electronic structure calculations on Au/STC/Au junctions: Density-functional-theory (DFT) calculations on the full solvated junction are computationally unpractical. To make the calculations feasible, we adopted a QM/MM approach where the full STC protein and the two gold electrodes, modeled as two mono-layer thick gold slabs, were treated at the DFT level. We checked in our previous work that the minimalistic two mono-layer thick gold slabs reproduce the Au density of states of a six-layer gold slab reasonably well [7]. The solvent water and the counter ions were modeled at the classical force field (MM) level. A problem of this setup is that the STC protein carries a net charge that would be treated at the DFT level while the compensating charge due to the counterions would be treated at the classical force field level. It is well known that the treatment of net charged subsystems at different levels of theories leads to artifacts and should be avoided. Thus, for the DFT calculations on the QM system, we changed the charge state of the amino acids back to the one in vacuum by protonation or deprotonation while keeping all other atomic positions unchanged [7,22]. In keeping with our previous work, both heme-cofactor propionate groups were protonated in the all-reduced STC structure, while one of them was kept deprotonated on each cofactor in the all-oxidized structure. This ensured charge neutrality in each of the QM and MM subsystems. The electrostatic interactions of the excess charge of the amino acids that are ionized in an aqueous solution (charged minus neutral) and the electrostatic interactions of the solvent water and counterions with the DFT subsystem are included in terms of an external Coulomb potential in the DFT calculations, as detailed in the section “External electrostatic potential in QM/MM calculations”. For a given junction structure (**L1**, **L2**, **S1**, and **S2**) and protein redox state, the external electrostatic potential in the QM region due to the excess charge of amino

acids, counterions, and the solvent was sampled on the time scale of the experimental electron transit time between the two electrodes, 128 ps, while keeping the STC protein fixed. The thermal average of the electrostatic potential over this time period was used as the effective external electrostatic potential in the QM/MM calculations of Kohn-Sham states and current-voltage calculations detailed below.

Following the setup from previous work [7,22], we used the PBE exchange-correlation potential [31], GTH pseudopotentials [32], and the DZVP-MOLOPT-SR basis set, as implemented in the CP2K software package [33], to obtain Kohn-Sham orbitals of the full junction using the QM/MM setup described above. Surface periodic boundary conditions along the gold slab planes (x, y directions) were applied, while the cluster boundary conditions were used in the junction direction (z) perpendicular to the surfaces. Electrostatic interactions were evaluated on the grid specified by a 500 Ry electron density cutoff, and the electronic-state occupation numbers were smeared by Fermi distribution with the effective electronic temperature 300 K. The wavefunction was obtained iteratively by an SCF procedure with the convergence criteria set to 10^{-6} a.u.

The Kohn-Sham (KS) one-electron states obtained from the QM/MM calculations were localized on the protein and the two gold slabs using the POD method [34,35]. The latter method transforms the KS Hamiltonian to a block structure:

$$\tilde{H} = \begin{bmatrix} \tilde{H}_{LL} & \tilde{H}_{LP} & \tilde{H}_{LR} \\ \tilde{H}_{PL} & \tilde{H}_{PP} & \tilde{H}_{PR} \\ \tilde{H}_{RL} & \tilde{H}_{RP} & \tilde{H}_{RR} \end{bmatrix}, \qquad (1)$$

where L, R, and P stand for the left electrode (bottom surface), right electrode (upper surface), and protein, respectively. Eigenvalues of the diagonal blocks are the localized-state energies $\varepsilon_{\alpha,j}$ for $\alpha \in \{L, R, P\}$, while the corresponding transformed off-diagonal block elements represent the electronic couplings between the localized states in the different regions.

Here a note regarding the alignment of the electronic energy levels of the metal and the protein electronic states is in order. The STM experiments are carried out without an electrochemical reference electrode, hence the electrochemical potential and the workfunction of the polycrystalline gold electrodes and thus the position of the heme redox levels with respect to the Fermi levels are not further known. However, experiments indicate that the Fermi levels of the electrodes are outside the redox potential window of the heme groups. In the absence of any further experimental information, we apply the same strategy to correct the DFT energy level offset between metallic and protein electronic states as in our previous work for the junctions in vacuum [7,22,38], that is, we apply the DFT+Σ scheme [36,37]. The PBE states from the POD protein blocks, $\varepsilon_{P,j}$, are shifted based on their occupancy:

$$\varepsilon_{\Sigma_j} = \varepsilon_{P,j} + \Sigma_{P,j} \qquad (2)$$

where $\Sigma_{P,j} = \Sigma^{0}_{P,j} + \Sigma^{pol}_{P,j}$ is comprised of two parts, the self-interaction error correction ($\Sigma^{0}_{P,j}$) and the image-charge interaction correction on the metal interface ($\Sigma^{pol}_{P,j}$). For all occupied states (j

= occ), we apply $\Sigma^0_{P,occ} = \Sigma^0_{P,HOMO}$ = -1.276 eV and for all unoccupied states (*j* = unocc) $\Sigma^0_{P,unocc} = \Sigma^0_{P,LUMO}$ = 1.525 eV. These values were obtained for all-oxidized STC using the optimally-tuned range-separated hybrid (OT-RSH) functional [39,40] for the heme cofactors in the gas phase (see Table S2 in SI of [7] for details) [7,22]. We apply the same corrections on the all-reduced STC structures in accord with [7,22]. The polarization contributions $\Sigma^{pol}_{P,j}$ were obtained by integration of the occupied $Fe^{2/3+}$ t_{2g} and unoccupied e_g molecular orbitals of the all-oxidized STC within the junction image-charge potentials of the **L1**, **L2**, **S1**, and **S2** structures, $\Sigma^{pol}_{P,occ} = \Sigma^{pol}_{Fe,t_{2g}}$ and $\Sigma^{pol}_{P,unocc} = \Sigma^{pol}_{Fe,eg}$. These values are summarized in Table S3 together with the final $\Sigma_{P,occ}$, $\Sigma_{P,unocc}$ corrections for the occupied/unoccupied states, respectively. As a result, the HOMO level of the protein is about -1.2 eV below the Fermi level of the electrodes, and the LUMO level is about 1.5 eV above the Fermi level, which is similar to vacuum structures and in qualitative agreement with experimental conclusions that transport is in the off-resonant regime [9]. While the presence of solvent water is likely to affect these corrections, we have verified that the computed currents and, in particular, the computed exponential distance decay factors β, are not very sensitive to the energy level offset within some reasonable bounds (see Table S4). For instance, if the energy level offset in the all-oxidized state of **L1** is changed by +0.5 (-0.5) eV with respect to the above value of -1.2 eV, the changes in current at 0.1 V bias are less than a factor of 1.5, about +30% (-19%). The effect on β values is negligible.

External electrostatic potential in QM/MM calculations: The external Coulomb potential in the QM region due to MM atoms was calculated using the smooth particle-mesh Ewald method (PME) [26,17] employing the 3D periodic boundary conditions used in the MD simulation above. The electrostatic potential generated by the MM region is calculated at every grid point of the QM box. A fine grid with a spacing of 0.7 Å was employed. The reciprocal space Ewald sum was computed via the *'pmepot'* VMD plugin [41], while the real space sum, Eq (3), was calculated using an in-house written code:

$$\phi_{dir}(r) = \sum_{n}\sum_{i=1}^{N}\frac{q_i erfc(\eta|r - r_i + n|)}{|r - r_i + n|} \quad (3)$$

We employed a real-space decay coefficient η = 0.26 $Å^{-1}$ and a real-space cutoff of 10 Å in our calculations. Water molecules are modeled using the TIP3P model [24], with partial charges q_i of -0.834 for oxygen atoms and 0.417 for hydrogen atoms. Sodium counterions have a charge of +1. The charge q_i on the atoms of the amino acid side chains that were protonated or deprotonated to obtain a neutral QM subsystem was set to equal the charge difference between the atomic charges in the charged and neutral states. Hence, this contribution models the effect of the excess charge of these amino acids, which is not explicitly included in the DFT calculation but modeled in terms of an electrostatic MM contribution.

To validate our method for calculating the electrostatic potential created by the solvent, we carried out a test calculation for a very large, periodically replicated box with dimensions 200 x 200 x 200 $Å^3$ containing 164 water molecules in the middle of the box and 182 Å vacuum in each direction. Due to the large vacuum layer, the contribution of water molecules in image

cells to the electrostatic potential in the central cell can be neglected, and the potential with and without periodic boundary conditions (PBC) will be the same. Hence, the electrostatic potential in the central cell obtained by direct Coulomb potential summation in cluster boundary conditions can be used to benchmark the accuracy of the Ewald summation in PBC. The box was divided into 500 grid points in each direction, resulting in a grid spacing of 0.4 Å. The maximum difference between the PBC and cluster-boundary calculations at any grid point did not exceed 0.6 mV indicating correct numerical implementation of the Ewald potential.

3. Tunneling current calculations

For the tunneling-current calculations, we apply the Landauer-Büttiker formalism [42,43], in which the current is obtained by integrating the transmission function over the Fermi window at a given bias potential:

$$I(V) = \frac{e}{\pi\hbar}\int T(E)[f_L(E,V) - f_R(E,V)]dE \tag{4}$$

The Fermi window is determined by the difference between the Fermi-Dirac distributions of the electronic-state occupancies in the left (f_L) and right (f_R) electrodes, respectively. The transmission function T is computed at zero bias in the Breit-Wigner approximation, assuming independent conduction channels represented by the individual localized molecular orbitals in the protein region:

$$T(E) = \sum_{j \in protein} \frac{\Gamma_j^{(L)}(E)\,\Gamma_j^{(R)}(E)}{\left(E - \varepsilon_{\Sigma_j}\right)^2 + \left[\Gamma_j^{(L)}(E) + \Gamma_j^{(R)}(E)\right]^2/4} \tag{5}$$

The $\Gamma_j^{(M)}$ functions are the so-called spectral densities (also known as interface couplings) of protein states j interacting with the electrode M = L, R. These functions are computed from the electrode-protein coupling elements $[H_{MP}]_{mj}$, which are weighted by the metallic densities of states ρ_M:

$$\Gamma_j^{(M)}(E) = 2\pi\left[\left|[H_{MP}]_{mj}\right|^2 \rho_M(\varepsilon_{M,m})\right]_{\varepsilon_{M,m} = E} \tag{6}$$

The computed tunneling current values for the reduced and oxidized **L1**, **L2**, **S1**, and **S2** junctions are collected in Table S4, where their dependence on the electronic-state offset $\Sigma_{P,occ}$ is shown. The corresponding conductance values are listed in Table S5.

Table S2: *Protonation states of the heme A and D propionate groups in the all-reduced and all-oxidized STC junction structures and their net charges in aqueous solution.*

State	Structure	Heme protonation				Net charge
		Heme -1	Heme-2	Heme-3	Heme-4	

Reduced	**L1**	A-/D-	A-/D-	A-/D-	A-/D-	-13
	**L2**	A-/D-	A-/D-	A-/DH	A-/D-	-13
	**S1**	A-/DH	A-/DH	A-/D-	A-/D-	-13
	**S2**	A-/D-	A-/D-	A-/D-	A-/D-	-14
Oxidized	**L1**	A-/D-	A-/D-	A-/D-	A-/D-	-9
	**L2**	A-/D-	A-/D-	A-/DH	A-/D-	-9
	**S1**	A-/DH	A-/DH	A-/D-	A-/D-	-9
	**S2**	A-/D-	A-/D-	A-/D-	A-/D-	-10

Table S3: *Interfacial band renormalization determined by image-charge interaction of iron t_{2g}/e_g band in the all-oxidized STC junctions. The values $\Sigma^{0}_{P,HOMO}$ = -1.276 eV and $\Sigma^{0}_{P,LUMO}$ = 1.525 eV, averaged over heme cofactors in all-oxidized vacuum STC junctions (c.f. Table S4 in SI of [7]), were applied to obtain the final corrections $\Sigma_{P,occ}$ and $\Sigma_{P,unocc}$ for the occupied/unoccupied states, respectively. All values are given in eV.*

	$\Sigma^{pol}_{Fe,t_{2g}}$	Σ^{pol}_{Fe,e_g}	$\Sigma_{P,occ}$	$\Sigma_{P,unocc}$
**L1**	0.082	-0.032	-1.194	1.493
**L2**	0.035	-0.027	-1.241	1.498
**S1**	0.059	-0.038	-1.217	1.487
**S2**	0.063	-0.055	-1.213	1.470
Average	0.060	-0.038	-1.216	1.487

Table S4: *Magnitudes of the computed tunneling currents I [nA] at 0.1 V bias for **L1**, **L2**, **S1**, and **S2** junction structures with all-reduced and all-oxidized STC, respectively, as functions of the occupied electronic-state offset $\Sigma_{P,occ}$ between the STC valence band maximum (HOMO) and electrode Fermi level E_f. The decay factors β [Å^{-1}], obtained by linear regression in the logarithm scale, are listed for each $\Sigma_{P,occ}$ value. The $\Sigma_{P,unocc}$ was shifted appropriately to keep the constant HOMO-LUMO gap as obtained by DFT+Σ.*

$\Sigma_{P,occ}$	Reduced STC					Oxidized STC				
	**L1**	**L2**	**S1**	**S2**	β	**L1**	**L2**	**S1**	**S2**	β
0.0	66.89	1.72	0.26	1.19	0.29	14.36	5.34	0.88	0.89	0.20
-0.1	17.55	1.60	0.25	1.11	0.23	6.84	4.29	0.66	0.79	0.18
-0.2	8.71	1.51	0.24	1.04	0.20	5.66	3.95	0.56	0.73	0.17
-0.3	7.74	1.43	0.23	0.98	0.20	5.20	3.68	0.50	0.68	0.18
-0.4	7.06	1.35	0.22	0.92	0.19	4.83	3.43	0.45	0.64	0.18
-0.5	6.48	1.29	0.21	0.87	0.19	4.51	3.21	0.41	0.61	0.18

-0.6	5.99	1.23	0.21	0.82	0.19	4.22	3.02	0.37	0.58	0.18
-0.7	5.55	1.17	0.20	0.78	0.19	3.98	2.84	0.34	0.55	0.18
-0.8	5.17	1.12	0.19	0.74	0.19	3.75	2.68	0.32	0.53	0.18
-0.9	4.83	1.07	0.19	0.71	0.18	3.55	2.54	0.30	0.51	0.18
-1.0	4.52	1.03	0.18	0.68	0.18	3.37	2.41	0.28	0.49	0.18
-1.1	4.25	0.99	0.18	0.65	0.18	3.21	2.28	0.27	0.47	0.18
-1.2	4.01	0.95	0.17	0.62	0.18	3.06	2.17	0.25	0.45	0.18
-1.3	3.79	0.91	0.16	0.59	0.18	2.92	2.07	0.24	0.43	0.18
-1.4	3.58	0.88	0.16	0.57	0.18	2.79	1.97	0.23	0.42	0.18
-1.5	3.40	0.85	0.16	0.55	0.18	2.67	1.88	0.22	0.41	0.18
-1.6	3.23	0.82	0.15	0.53	0.17	2.56	1.80	0.21	0.39	0.18
-1.7	3.08	0.79	0.15	0.51	0.17	2.46	1.72	0.20	0.38	0.18
-1.8	2.93	0.77	0.14	0.49	0.17	2.37	1.65	0.19	0.37	0.18
-1.9	2.80	0.74	0.14	0.47	0.17	2.28	1.58	0.18	0.36	0.18
-2.0	2.68	0.72	0.14	0.46	0.17	2.20	1.52	0.17	0.35	0.18

Table S5: *Computed conductance G ($G_0 = 7.748\times10^{-5}$ S units) for* ***L1***, ***L2***, ***S1***, *and* ***S2*** *junction structures of all-reduced and all-oxidized STC, respectively, as a function of the occupied electronic-state offset $\Sigma_{P,occ}$ between the STC valence band maximum (HOMO) and electrode Fermi level E_f.*

$\Sigma_{P,occ}$	Reduced STC				Oxidized STC			
	**L1**	**L2**	**S1**	**S2**	**L1**	**L2**	**S1**	**S2**
0.0	5.69E-3	2.15E-4	3.25E-5	1.51E-4	1.56E-3	7.22E-4	1.06E-4	1.11E-4
-0.1	3.07E-3	2.01E-4	3.13E-5	1.41E-4	9.49E-4	5.42E-4	8.48E-5	1.00E-4
-0.2	1.13E-3	1.89E-4	3.01E-5	1.32E-4	7.26E-4	4.96E-4	7.10E-5	9.24E-5
-0.3	9.85E-4	1.79E-4	2.90E-5	1.24E-4	6.66E-4	4.62E-4	6.28E-5	8.65E-5
-0.4	8.98E-4	1.70E-4	2.80E-5	1.17E-4	6.18E-4	4.31E-4	5.65E-5	8.16E-5
-0.5	8.24E-4	1.62E-4	2.70E-5	1.11E-4	5.77E-4	4.04E-4	5.14E-5	7.73E-5
-0.6	7.61E-4	1.54E-4	2.61E-5	1.05E-4	5.40E-4	3.79E-4	4.71E-5	7.36E-5
-0.7	7.05E-4	1.47E-4	2.52E-5	9.98E-5	5.08E-4	3.57E-4	4.36E-5	7.02E-5
-0.8	6.56E-4	1.41E-4	2.44E-5	9.50E-5	4.80E-4	3.37E-4	4.05E-5	6.72E-5
-0.9	6.13E-4	1.35E-4	2.36E-5	9.06E-5	4.54E-4	3.19E-4	3.79E-5	6.44E-5
-1.0	5.75E-4	1.30E-4	2.29E-5	8.65E-5	4.31E-4	3.02E-4	3.56E-5	6.19E-5
-1.1	5.40E-4	1.24E-4	2.22E-5	8.27E-5	4.10E-4	2.87E-4	3.36E-5	5.95E-5
-1.2	5.09E-4	1.20E-4	2.15E-5	7.92E-5	3.91E-4	2.73E-4	3.17E-5	5.73E-5
-1.3	4.81E-4	1.15E-4	2.09E-5	7.59E-5	3.73E-4	2.60E-4	3.01E-5	5.53E-5
-1.4	4.55E-4	1.11E-4	2.03E-5	7.29E-5	3.57E-4	2.48E-4	2.86E-5	5.34E-5
-1.5	4.31E-4	1.07E-4	1.97E-5	7.00E-5	3.41E-4	2.36E-4	2.73E-5	5.16E-5
-1.6	4.10E-4	1.03E-4	1.92E-5	6.73E-5	3.27E-4	2.26E-4	2.61E-5	5.00E-5
-1.7	3.90E-4	9.97E-5	1.86E-5	6.48E-5	3.14E-4	2.16E-4	2.49E-5	4.84E-5
-1.8	3.72E-4	9.64E-5	1.81E-5	6.25E-5	3.02E-4	2.07E-4	2.39E-5	4.69E-5
-1.9	3.55E-4	9.32E-5	1.76E-5	6.03E-5	2.91E-4	1.99E-4	2.29E-5	4.55E-5
-2.0	3.40E-4	9.03E-5	1.72E-5	5.82E-5	2.80E-4	1.91E-4	2.20E-5	4.42E-5

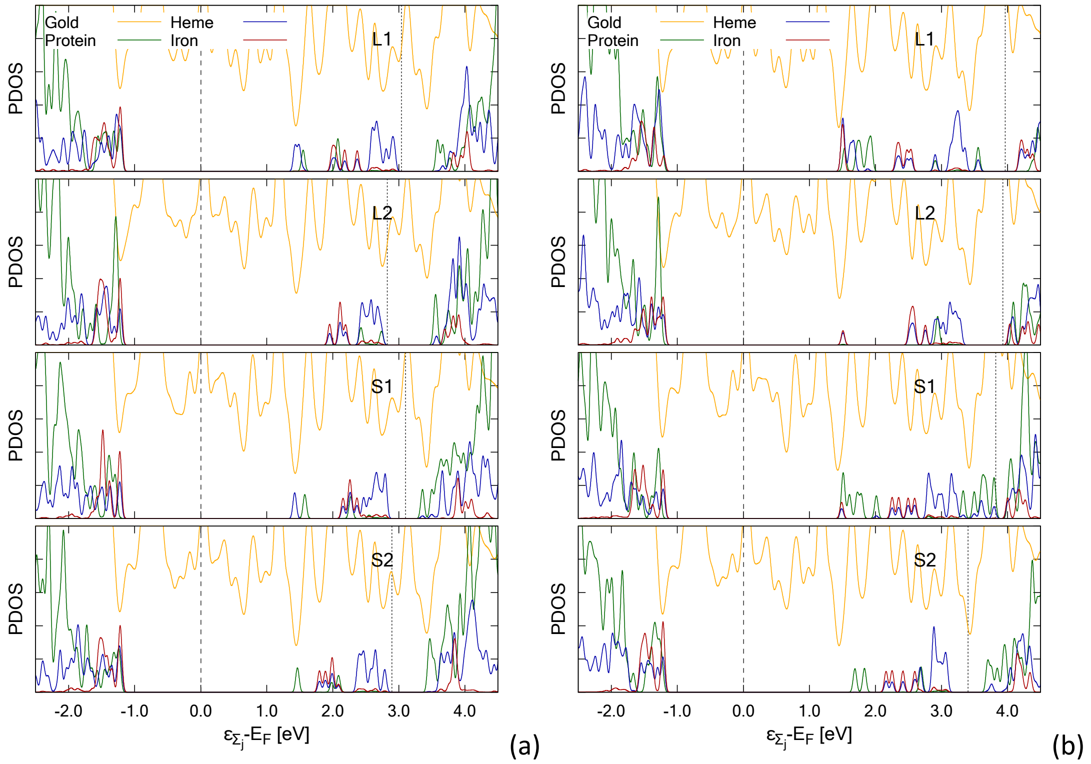

Figure S7: *Projected densities of states (PDOS) of (a) all-reduced and (b) all-oxidized **L1**, **L2**, **S1**, and **S2** junction structures broken down in electronic state contributions from gold (orange), protein amino acids (green), bis-histidine heme cofactors excluding iron (blue), and iron (red). An offset of* $\Sigma_{P,occ}$*= -1.2 eV and* $\Sigma_{P,unocc} = +1.5\ eV$ *is applied for the occupied and unoccupied electronic states of the protein energy levels relative to the electrode Fermi level* E_f *for the all-reduced / all-oxidized protein. The energy states are plotted relative to* E_f *(indicated by the vertical dashed line). The unoccupied-state threshold for the tunneling-current calculation is shown by the vertical dotted lines.*

4. Experimental and theoretical I(V) characteristics and SDS-PAGE analysis of the protein.

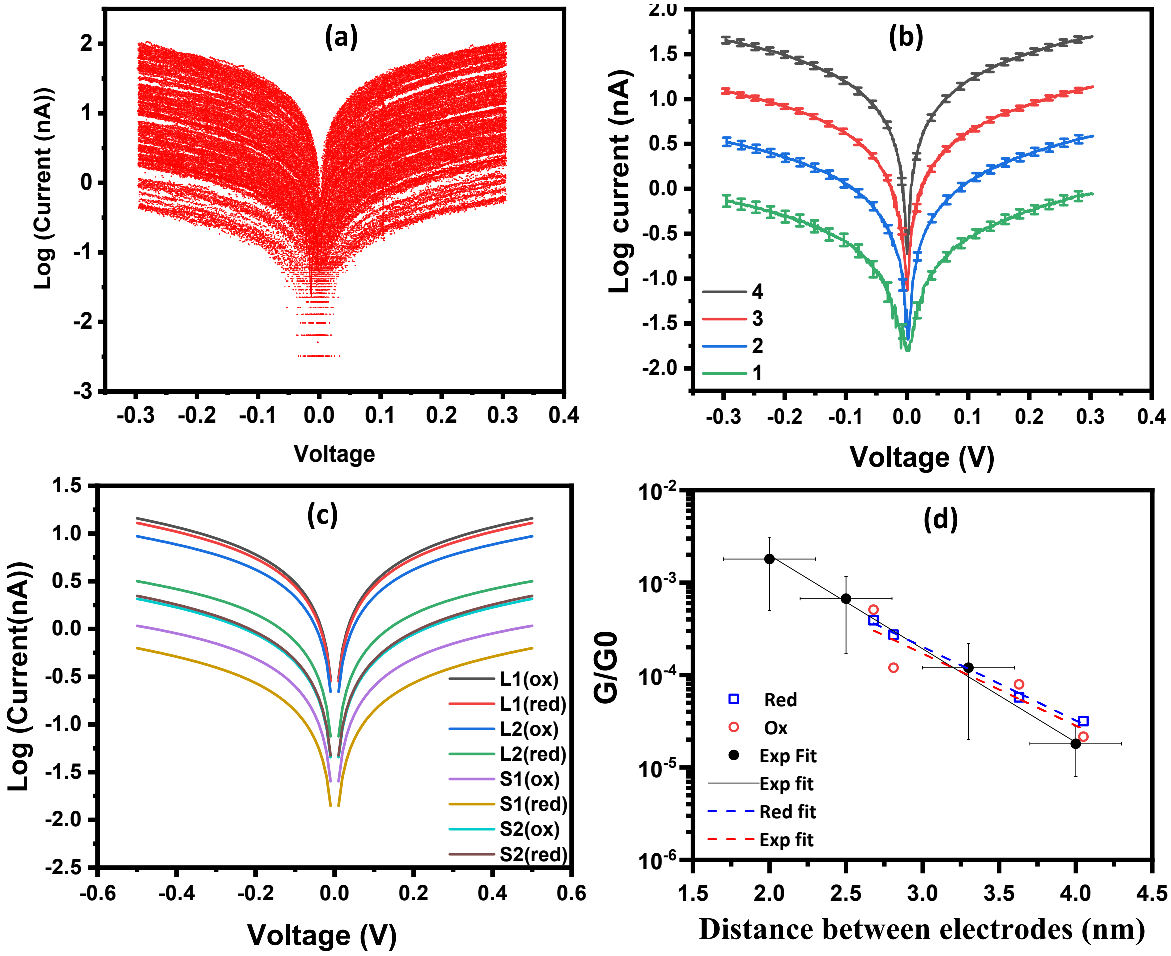

Figure S8: *(a) All I-V curves measured at different tip distances plotted together, showing the four different conductance regions. (b) Averaged I-V curves measured at different tip distances, error bars are shown as the standard deviation of the I-V curves measured at different distances (2.0±0.3, 2.5±0.3, 3.3±0.3 and 4.0±0.3 nm), where 1, 2, 3 and 4 represents the number of hemes probed in the junction. (c) Theoretically simulated I-V curves for **L1**(ox), **L1**(red), **L2**(ox), **L2**(red), **S1**(ox), **S1**(red), **S2**(ox) and **S2**(red). (d) Semi-log conductance (G/G_0) decay plot representing STC conductance extracted from the computed I-V curves against the electrode-electrode junction gap separation. Best fits give decay constants $\beta = 1.84$ nm^{-1} and 1.80 nm^{-1} for all-oxidized and all-reduced STC, respectively.*

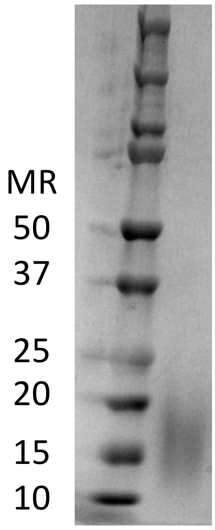

Figure S9: *X: SDS-PAGE gel (Coomassie stained) of S87C STC variant. To obtain monomeric forms, 1 mM TCEP was added to samples prior to loading. Molecular Weight (MR) values for the protein ladder are kDa.*

5. Experimental data comparing across different MHC proteins.

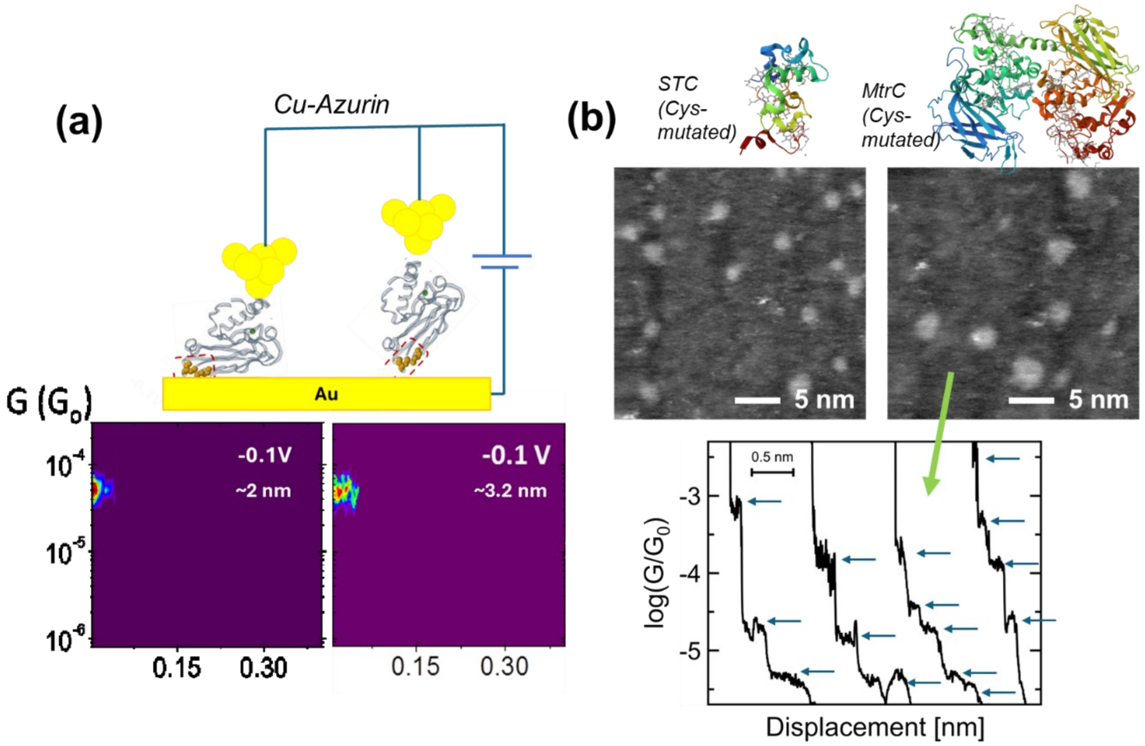

Figure S10: *(a) Fixed electrode-electrode gap charge transport data for a soluble Cu-Azurin whose redox cofactor is well buried within the peptide structure, showing conductance invariance as a function of the electrode-electrode gap separation. (b) Dynamic charge transport data (bottom panel) and STM image comparison to STC (top panel) for a soluble*

decaheme MtrC, showing consistent protein size and increased number of observed conductance states in the individual conductance traces.

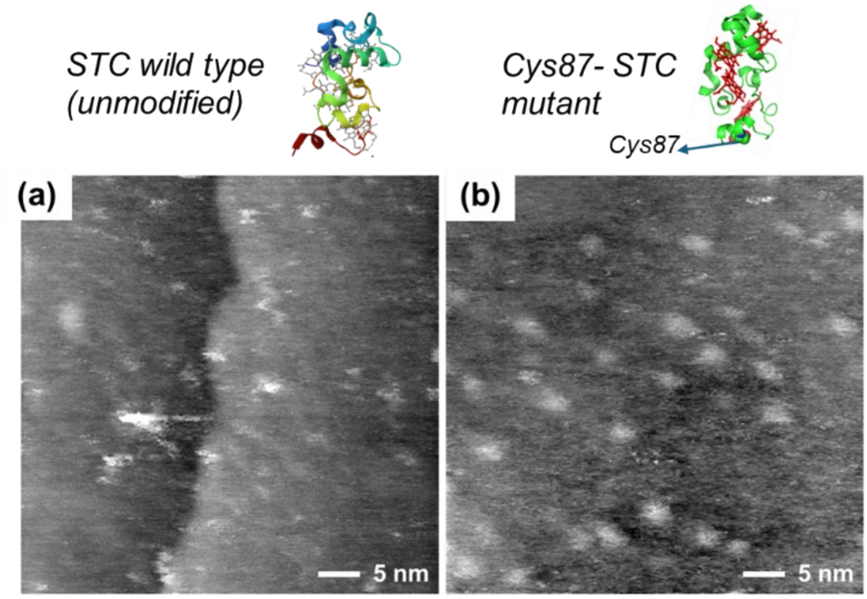

Figure S11: *In situ STM images for (a) a non-modified STC and (b) a surface-Cys modified STC.*

References

[1] van Wonderen, J. H. et al. Photosensitised Multiheme Cytochromes as Light-Driven Molecular Wires and Resistors. ChemBioChem 19, 2206–2215 (2018).

[2] Garg, K. et al. Direct evidence for heme-assisted solid-state electronic conduction in multi-heme c -type cytochromes. Chem. Sci. (2018) doi:10.1039/C8SC01716F.

[3] Xu, B. & Tao, N. J. Measurement of single-molecule resistance by repeated formation of molecular junctions. Science 301, 1221–3 (2003).

[4] Artés, J. M., Díez-Pérez, I. & Gorostiza, P. Transistor-like behavior of single metalloprotein junctions. Nano Lett. 12, 2679–2684 (2012).

[5] Aragonès, A. C. et al. Highly conductive single-molecule wires with controlled orientation by coordination of metalloporphyrins. Nano Lett. 14, 4751–6 (2014).

[6] Fraczkiewicz, R. & Braun, W. Exact and efficient analytical calculation of the accessible surface areas and their gradients for macromolecules. J. Comput. Chem. 19, 319–333 (1998).

[7] Futera, Z.; Wu, Z.; Blumberger, J.: Tunneling-to-Hopping Transition in Multiheme Cytochrome Bioelectronic Junctions. *J. Chem. Phys. Lett.* **14**, 445-452 (2023).

[8] Leys, D.; Meyer, T. E.; Tsapin, A. S.; Nealson, K. H.; Cusanovich, M. A.; Van Beeumen, J. J.: Crystal Structures at Atomic Resolution Reveal the Novel Concept of “Electron-Harvesting” as a Role for the Small Tetraheme Cytochrome c. *J. Biol. Chem.* **277**(38), 35708-35711 (2002).

[9] Garg, K.; Ghosh, M.; Eliash, T.; van Wonderen, J. H.; Butt, J. N.; Shi, L.; Jian, X.; Futera, Z.; Blumberger, J.; Pecht, I.; Sheves, M.; Cahen, D.: Direct Evidence for Heme-Assisted Solid-State Electronic Conduction in Multi-Heme c-Type Cytochromes. *Chem. Sci.* **9**, 7304-7310 (2018).

[10] Sondergaard, C. R.; Olsson, M. H. M.; Rostkowski, M.; Jensen, J. H.: Improved Treatment of Ligands and Coupling Effects in Empirical Calculation and Rationalization of pKa Values. *J. Chem. Theory Comput.* **7**(7), 2284-2295 (2011).

[11] Olsson, M. H. M; Sondergaard, C. R.; Rostkowski, M.; Jensen, J. H.: PROPKA3: Consistent Treatment of Internal and Surface Residues in Empirical pKa Predictions. *J. Chem. Theory Comput.* **7**(2), 525-537 (2011).

[12] Iori, F.; Di Felice, R.; Molinari, E.; Corni, S.: GolP: An Atomistic Force-Field to Describe the Interaction of Proteins with Au(111) Surfaces in Water. *J. Comput. Chem.* **30**, 1465-1476 (2009).

[13] Wright, L. B.; Rodger, P. M.; Corni, S.; Walsh, T. R.: GolP-CHARMM: First-Principles Based Force Fields for the Interaction of Proteins with Au(111) and Au(100). *J. Chem. Theory Comput.* **9**, 1616-1630 (2013).

[14] Futera, Z.; Blumberger, J.: Adsorption of Amino Acids on Gold: Assessing the Accuracy of the GolP-CHARMM Force Field and Parametrization of Au-S Bonds. *J. Chem. Theory Comput.* **15**, 613-624 (2019)

[15] MacKerell, A. D., Jr.: All-Atom Empirical Potential for Molecular Modeling and Dynamics Studies of Proteins. *J. Phys. Chem. B* **102**, 3586-3616 (1998).

[16] MacKerell, A. D., Jr.; Feig, M.; Brooks, C. L., III.: Extending the Treatment of Backbone Energetics in Protein Force Fields: Limitations of Gas-Phase Quantum Mechanics in Reproducing Protein Conformational Distributions in Molecular Dynamics Simulations. *J. Comput. Chem.* **25**, 1400-1415 (2004).

[17] Breuer, M.; Zarzycki, P.; Blumberger, J.; Rosso, K.: Thermodynamics of Electron Flow in the Bacterial Deca-Heme Cytochrome MtrF. *J. Am. Chem. Soc.* **134**(24), 9868-9871 (2012).

[18] Breuer, M.; Rosso, K. M.; Blumberger, J.: Electron Flow in Multiheme Bacterial Cytochromes is a Balancing Act Between Heme Electronic Interaction and Redox Potentials. *Proc. Natl. Acad. Sci. U.S.A.* **111**(2), 611-616 (2014).

[19] Jiang, X.; Futera, Z.; Ali, Md. E.; Gajdos, F.; von Rudorff, G. F.; Carof, A.; Breuer, M.; Blumberger, J.: Cysteine Linkages Accelerate Electron Flow through Tetra-Heme Protein STC. *J. Am. Chem. Soc.* **139**, 17237-17240 (2017).

[20] Jiang, X.; Burger, B.; Gajdos, F.; Bortolotti, C.; Futera, Z.; Breuer, M.; Blumberger, J.: Kinetics of Trifurcated Electron Flow in the Decaheme Bacterial Proteins MtrC and MtrF. *Proc. Natl. Acad. Sci. U.S.A.* **116**(9), 3425-3430 (2019).

[21] van Wonderen, J. H.; Hall, Ch. R.; Jiang, X.; Adamczyk, K.; Carof, A.; Heisler, I.; Piper, S. E. H.; Clarke, T. A.; Watmough, N. J.; Sazanovich, I. V.; Towrie, M.; Meech, S. R.; Blumberger, J.; Butt, J. N.: Ultrafast Light-Driven Electron Transfer in a Ru(II)tris(bipyridine)-Labeled Multiheme Cytochrome. *J. Am. Chem. Soc.* **141**, 15190-15200 (2019).

[22] Futera, Z.; Ide, I.; Kayser, B.; Garg, K.; Jiang, X.; van Wonderen J. H.; Butt, J. N.; Ishii, H.; Pecht, I.; Sheves, M.; Cahen, D.; Blumberger, J.: Coherent Electron Transport across a 3 nm Bioelectronic Junction Made of Multi-Heme Proteins. *J. Phys. Chem. Lett.* **11**, 9766-9774 (2020).

[23] van Wonderen, J. H.; Adamczyk, K.; Wu, X.; Jiang, X.; Piper, S. E. H.; Hall, Ch. R.; Edwards, M. J.; Clarke, T. A.; Zhang, H.; Jeuken, L. J. C.; Sazanovich, I. V.; Towrie, M.; Blumberger, J.; Meech, S. R.; Butt, J. N.: Nanosecond Heme-to-Heme Electron Transfer Rates in a Multiheme Cytochrome Nanowire Reported by a Spectrally Unique His/Met-Ligated Heme. *Proc. Natl. Acad. Sci. U.S.A.* **118**(39), e2107939118 (2021).

[24] Jorgensen, W. L. and Chandrasekhar, J.; Madura, J. D.; Impley, R. W.; Klein, M. L.: Comparison of Simple Potential Functions for Simulating Liquid Water. *J. Chem. Phys.* **79**(2), 926-935 (1983).

[25] Abraham, M. J.; Murtola, T.; Schulz, R.; Pall, S.; Smith, J. C.; Hess, B.; Lindahl, E.: GROMACS: High Performance Molecular Simulations through Multi-Level Parallelism from Laptops to Supercomputers. *SoftwareX* **1-2**, 19-25 (2015).

[26] Darden, T.; York, D.; Pedersen, L.: Particle Mesh Ewald: An N log N Method for Ewald Sums in Large Systems. *J. Chem. Phys.* **98**, 10089-10092 (1993).

[17] Essmann, U.; Perera, L.; Berkowitz, M. L.; Darden, T.; Lee, H.; Pedersen, L. G.: A Smooth Particle Mesh Ewald Method. *J. Chem. Phys.* **103**, 8577-8593 (1995).

[28] Nose, S.: A Unified Formulation of the Constant Temperature Molecular Dynamics Methods. *J. Chem. Phys.* **81**, 511-519 (1984).

[29] Hoover, W. G.: Canonical Dynamics: Equilibrium Phase-Space Distribution. *Phys. Rev. A: At., Mol., Opt. Phys.* **31**, 1695-1697 (1985).

[30] Biriukov, D.; Futera, Z.: Adsorption of Amino Acids at the Gold/Aqueous Interface: Effect of an External Electric Field. *J. Phys. Chem. C* **125**, 7856-7867 (2021).

[31] Perdew, J. P.; Burke, K.; Ernzerhof, M.: Generalized Gradient Approximation Made Simple. *Phys. Rev. Lett.* **77**(18), 3865-3868 (1996).

[32] Goedecker, S.; Teter, M.; Hutter, J.: Separable Dual-Space Gaussian Pseudopotential. *Phys. Rev. B* **54**(3), 1703-1710 (1996).

[33] Hutter, J.; Iannuzzi, M.; Schiffmann, F.; VandeVondele, J.: CP2K: Atomistic Simulations of Condensed Matter Systems. *WIREs Comput. Mol. Sci.* **4**, 15-25 (2014).

[34] Kondov, I.; Cizek, M.; Benesch, C.; Wang, H.; Thoss, M.: Quantum Dynamics of Photoinduced Electron-Transfer Reactions in Dye-Semiconductor Systems: First-Principles Description and Application to Coumarin 343-TiO_2. *J. Phys. Chem. C* **111**, 11970-11981 (2007).

[35] Futera, Z.; Blumberger, J.: Electronic Couplings for Charge Transfer across Molecular/Metal and Molecule/Semiconductor Interfaces: Performance of the Projector Operator-Based Diabatization Approach. *J. Phys. Chem. C* **121**, 19677-19689 (2017).

[36] Neaton, J. B.; Hybertsen, M. S.; Louie, S. G.: Renormalization of Molecular Electronic Levels at Metal-Molecule Interfaces. *Phys. Rev. Lett.* **97**, 216405 (2006).

[37] Egger, D. A.; Liu, Z.-F.; Neaton, J. B.; Kronik, L.: Reliable Energy Level Alignment at Physisorbed Molecule-Metal Interfaces from Density Functional Theory. *Nano Lett.* **15**, 2448-2455 (2015).

[38] Futera, Z.: Amino-Acid Interactions with the Au(111) Surface: Adsorption, Band Alignment, and Interfacial Electronic Coupling. *Phys. Chem. Chem. Phys.* **23**, 10257-10266 (2021).

[39] Baer, R.; Livshits, E.; Salzner, U.: Tuned Range-Separated Hybrids in Density Functional Theory. *Ann. Rev. Phys. Chem.* **61**, 85-109 (2010).

[40] Stein, T.; Eisenberg, H.; Kronik, L.; Baer, R.: Fundamental Gaps in Finite Systems from Eigenvalues of a Generalized Kohn-Sham Method. *Phys. Rev. Lett.* **105**, 266802 (2010).

[41] Aksimentiev, A.; Schulten, K.: Imaging α-Hemolysin with Molecular Dynamics: Ionic Conductance, Osmotic Permeability, and the Electrostatic Potential Map. *Biophys. J.* **88** (6), 3745-3761 (2005).

[42] Landauer, R.: Spatial Variation of Currents and Fields Due to Localized Scatterers in Metallic Conduction. *IBM J. Res. Dev.* **1**, 233 (1957).

[43] Buttiker, M.: Four-Terminal Phase-Coherent Conductance. *Phys. Rev. Lett.* **57**(14), 1761-1764 (1986).
